# Cdc42-Specific GTPase-Activating Protein Rga1 Squelches Crosstalk between the High-Osmolarity Glycerol (HOG) and Mating Pheromone Response MAPK Pathways

**DOI:** 10.3390/biom11101530

**Published:** 2021-10-17

**Authors:** Jesse C. Patterson, Louise S. Goupil, Jeremy Thorner

**Affiliations:** 1 Department of Molecular and Cell Biology, Division of Biochemistry, Biophysics and Structural Biology, University of California, Berkeley, CA 94720, USA; 2 Yaffe Laboratory, David H. Koch Institute for Integrative Cancer Research, Massachusetts Institute of Technology, Cambridge, MA 02139, USA; jessep@mit.edu; 3 Harney Science Center, Department of Biology, University of San Francisco, Rm. 258, San Francisco, CA 94117, USA; lgoupil@usfca.edu

**Keywords:** protein phosphorylation, single-cell analysis, cell regulation, transcriptional, reporters, signal insulation

## Abstract

Eukaryotes utilize distinct mitogen/messenger-activated protein kinase (MAPK) pathways to evoke appropriate responses when confronted with different stimuli. In yeast, hyperosmotic stress activates MAPK Hog1, whereas mating pheromones activate MAPK Fus3 (and MAPK Kss1). Because these pathways share several upstream components, including the small guanosine-5'-triphosphate phosphohydrolase (GTPase) cell-division-cycle-42 (Cdc42), mechanisms must exist to prevent inadvertent cross-pathway activation. Hog1 activity is required to prevent crosstalk to Fus3 and Kss1. To identify other factors required to maintain signaling fidelity during hypertonic stress, we devised an unbiased genetic selection for mutants unable to prevent such crosstalk even when active Hog1 is present. We repeatedly isolated truncated alleles of *RGA1*, a Cdc42-specific GTPase-activating protein (GAP), each lacking its C-terminal catalytic domain, that permit activation of the mating MAPKs under hyperosmotic conditions despite Hog1 being present. We show that Rga1 down-regulates Cdc42 within the high-osmolarity glycerol (HOG) pathway, but not the mating pathway. Because induction of mating pathway output via crosstalk from the HOG pathway takes significantly longer than induction of HOG pathway output, our findings suggest that, under normal conditions, Rga1 contributes to signal insulation by limiting availability of the GTP-bound Cdc42 pool generated by hypertonic stress. Thus, Rga1 action contributes to squelching crosstalk by imposing a type of “kinetic proofreading”. Although Rga1 is a Hog1 substrate in vitro, we eliminated the possibility that its direct Hog1-mediated phosphorylation is necessary for its function in vivo. Instead, we found first that, like its paralog Rga2, Rga1 is subject to inhibitory phosphorylation by the *S. cerevisiae* cyclin-dependent protein kinase 1 (Cdk1) ortholog Cdc28 and that hyperosmotic shock stimulates its dephosphorylation and thus Rga1 activation. Second, we found that Hog1 promotes Rga1 activation by blocking its Cdk1-mediated phosphorylation, thereby allowing its phosphoprotein phosphatase 2A (PP2A)-mediated dephosphorylation. These findings shed light on why Hog1 activity is required to prevent crosstalk from the HOG pathway to the mating pheromone response pathway.

## 1. Introduction

Multiple MAPK pathways enable a eukaryotic cell to respond appropriately when exposed to diverse stimuli. These signaling cascades often share multiple components [1,2], raising the potential for inappropriate cross-wiring and degradation of signaling specificity [3,4,5]. Such crosstalk often occurs only under specific conditions [6,7], and understanding the extent of signaling pathway interaction and its consequences remains a major challenge [8,9].

When cells of the budding yeast (*Saccharomyces cerevisiae*) are subjected to hypertonic conditions, the MAPK Hog1 is activated via either of two routes that converge on an upstream MAPK kinase (MAPKK) Pbs2 [10,11,12] (Figure S1, Panel A). The Sln1 branch resembles two-component phosphorelay systems prominent in prokaryotes. The Sho1 branch, by contrast, has a canonical MAPK pathway architecture. At its apex is the integral plasma membrane (PM) protein Sho1 [13], which possesses four membrane-spanning segments and a C-terminal SH3 domain. Sho1 forms complexes with three highly *O*-glycosylated, single-pass transmembrane proteins (Hkr1, Msb2, and Opy2) [14,15]. Upon hyperosmotic stress, these osmosensors actuate production of the GTP-bound state of Cdc42, a small (21 kDa) PM-anchored Ras family guanosine-5′-triphosphate phosphohydrolase (GTPase), by an as yet unclear mechanism (but, likely involving localized recruitment and/or activation of the Cdc42-specific guanine nucleotide exchange factor (GEF) Cdc24 [16,17,18]). The GTP-bound Cdc42 so generated has at least two direct effectors. First, Cdc42-GTP associates with Ste50 [19], a small adaptor protein that is a tightly bound non-catalytic subunit of a MAPKK kinase (MAPKKK) Ste11 [20,21]. Ste50 and Ste11 are, in turn, tethered to other components of the Sho1-associated PM complex [22,23,24,25], including a scaffold protein (Ahk1) that also associates with Sho1, Hkr1, and the MAPKK Pbs2 [26]. Second, Cdc42-GTP binds to and switches on the activity of the p21-activated protein kinase (PAK) Ste20 [27,28,29]. By this means, activated Ste20 is able to encounter, phosphorylate, and thereby activate Ste11 [30], which, in turn, phosphorylates and activates Pbs2 [31]. Once activated, Pbs2, which is also tightly bound to Sho1 itself [32,33], phosphorylates and activates the Hog1 MAPK [12,34]. Activated Hog1 reportedly delays cell cycle progression in G1 [35,36], in S phase [37], in G2 [38], and during mitotic exit [39]; but, most importantly, activated Hog1 greatly accelerates production and accumulation of intracellular glycerol to act as the osmolyte to counter-balance the challenge of external hypertonic conditions [40], a metabolic switch that is required for yeast cell survival when subjected to hyperosmotic shock [41,42,43].

When haploid *S. cerevisiae* cells are exposed to the peptide mating pheromone of the opposite mating type (e.g., exposure of *MAT***a** cells to α-factor) (Figure S1, Panel B), the MAPK Fus3 is activated. This response is initiated by pheromone binding to a cognate G-protein-coupled receptor (GPCR) [1,44,45]. Upon receptor occupancy, the released, but membrane-anchored, Gβγ complex recruits Ste20 via interaction with a motif in its C-terminus [46] and also locally generates active Cdc42-GTP through recruitment of Cdc24 via an adaptor protein, Far1 [47], which also serves as a CDK inhibitor [48,49]. Gβγ also recruits the scaffold protein Ste5, which carries as passengers the MAPKKK Ste11, the MAPKK Ste7, and the MAPK Fus3 [50]. Thus, a nexus is created that allows for efficient Ste20-mediated activation of the cascade that generates activated Fus3. The Cdc42–Ste20–Ste50–Ste11–Ste7 complex also functions to activate the MAPK Kss1 of the filamentous growth pathway [17,51], but does so in a Gβγ- and Ste5-independent manner because Kss1 does not bind to this scaffold protein [52,53]. Activated Fus3 causes cell cycle arrest in G1, polarized growth (shmoo formation), and induces expression of gene products that confer the capacity to fuse with a cell of the opposite mating type (conjugation) [44,54].

Although Cdc42, Ste50, Ste11, and Ste20 are all necessary for activation of both Hog1 and Fus3, hyperosmotic stress results only in Hog1 phosphorylation, and exposure to pheromone results only in Fus3 phosphorylation [55]. Revealingly, however, in the absence of Hog1 (or Pbs2) [56], or simply by inhibiting Hog1 kinase activity [57], hyperosmotic stress causes activation of Fus3 (and Kss1) in a manner that depends on all of upstream components shared by the HOG and mating pathways. Therefore, Hog1 plays an active role in preventing crosstalk. Although severe hyperosmotic stress slightly delays and modestly attenuates mating pathway output [55,58], Hog1 action does not prevent crosstalk through outright inhibition of any specific stage of the mating pathway per se. Instead, our prior work [55] has shown that Hog1 action prevents upstream factors activated by hypertonic stress from productively interacting with the mating pathway, a mechanism for imposing accuracy in signaling termed “insulation” [55,59,60]. As shown previously by others [33,56], only the Sho1 branch (and not the Sln1 branch) of the HOG pathway is capable of eliciting crosstalk to the mating pheromone response pathway when the function of Hog1 is compromised. How activation of pathways containing shared components is channeled to the appropriate output, depending on the context of the initial stimulus, is a critical question to be answered to understand the molecular basis of MAPK signaling fidelity.

As an approach to address this specific issue, we reasoned that the requirement for active Hog1 to block crosstalk indicates that its action likely stimulates, either directly or indirectly, a process that inhibits crosstalk. If so, then we might be able to identify factors that participate in inhibiting crosstalk by finding mutants in which hyperosmotic stress elicits mating pathway activation even though active Hog1 is present. Moreover, such mutants should only display crosstalk when Sho1 is present. Toward that end, we devised a genetic selection by constructing a yeast strain that carried two functional copies of both Hog1 and Pbs2, to ensure that mutations defective in preventing crosstalk, if they exist, would be isolated in other genes.

Applying this genetic selection repeatedly yielded mutations in the same locus and the mutant alleles were, in every case, truncations that removed the C-terminal end of the gene product. These attributes indicated, first, that the protein encoded by this gene contributes to the insulation mechanism that prevents propagation of a signal from the hyperosmotic stress pathway to the pheromone response pathway. Second, the fact that every mutant allele generated an N-terminal fragment suggests that, at least in part, their phenotype also likely arises by virtue of these fragments acting in a dominant-negative fashion. The gene we identified encodes Rga1, a Cdc42-specific GTPase-activating protein (GAP). Here we describe our selection, the function of Rga1 in regulating the extent of Hog1 activation, and the role of Rga1 in suppressing crosstalk to the mating pathway. Moreover, illuminating how Hog1 activity is required to prevent crosstalk, we show that inhibitory cell cycle-dependent phosphorylation of Rga1 is alleviated in response to hyperosmotic stress in a Hog1-dependent manner, thereby promoting Rga1 activity.

## 2. Materials and Methods

### 2.1. Yeast Strains, Plasmids and Growth Conditions

Yeast strains (Supplementary Table S1) and plasmids (Supplementary Table S2) were constructed using standard molecular biology and genetic techniques [61]. The strain used for the genetic selection, YJP394, was constructed from YJP123. The *URA3*-marked *FUS1_prom_*-*HIS3* reporter from pDH106 was integrated at the *HIS3* locus, followed by switching its *URA3* marker to *LYS2* using the cassette in the marker conversion plasmid M2660. *FAR1* was replaced with *far1-T306A,* a mutant allele that cripples only its G1 arrest function [48,62], using plasmid pJT4319 and PCR-mediated one-step gene replacement. An extra copy of *HOG1-AS* and an extra copy of *PBS2* were integrated at the *LEU2* locus by amplification of a *HOG1-AS*::*PBS2*::*LEU2* fragment from plasmid PJT4320 using primers containing homology to the *LEU2* ORF, and functionally confirmed by complementation.

Unless otherwise indicated, yeast strains were grown overnight in synthetic complete dextrose (SCD) medium at 30 °C, reinoculated into fresh medium, and grown for 5 h to mid-exponential phase. The addition of stimulants or drugs was achieved by diluting the culture in an equal volume of medium containing twice the desired concentration of additive. Pheromone stimulation in synthetic medium was performed in glass tubes pre-treated with 1% bovine-serum albumin [55]. To induce *RGA1* overexpression (and avoid any complicating effects of a shift in carbon source), strains containing the tripartite *S. cerevisiae* Gal4–human estrogen receptor–herpes simplex virus transactivator VP16 fusion protein (Gal4–ER–VP16 or GEV) [63,64] and a *URA3*-marked *CEN* plasmid (pRS316) expressing *RGA1* from the *GAL1,10* promoter were grown to mid-exponential phase and then treated with β-estradiol (20 μM final concentration) for 2 h before further manipulation.

The *RGA1* gene from YPH499 [65] was used for all plasmid and strain constructs. Although both derived from S288C, the primary sequence of the 1007-residue Rga1 polypeptide YPH499 differs from that in BY4741 [66] at the following five positions: Glu for Asp457; Pro for Thr507; Ala for Val866; Arg for Lys898; and Gly for Ser926. These polymorphisms appear to have no functional consequences. We found C-terminally tagged Rga1-3XHA to be non-functional, as judged by mating pathway reporter activation upon Hog1-as inhibition under isosmotic conditions; hence, for in vivo experiments, only N-terminally tagged Rga1 fusions were used and found to be functional by the same criterion. pETGST3C was constructed by amplification of the coding sequence for glutathione-S-transferase from pGEX4T-1 (without the stop codon). The primers used added a *Vsp*I site to the 5′-end, and the protease 3C cleavage coding sequence as well as an *Nde*I site to the 3′-end. This amplicon then was ligated into the *Nde*I site of pET21a.

### 2.2. Genetic Selection for Mutants that Result in Crosstalk

The yeast strain YJP394 (carrying a *URA3*-marked *CEN* plasmid expressing *SHO1*) was grown overnight in 5 mL SCD-Ura medium and aliquots containing ≥10^7^ cells were spread on 30 SCD-Ura/-His plates containing 1 M sorbitol and 9 mM 3-aminotriazole (3-AT). From these, ~300 spontaneously-arising His^+^ mutants were purified by patching onto fresh SCD-Ura/-His + 1 M sorbitol + 9 mM 3-AT medium plates, replica plated to SCD medium, and then finally replica plated to SCD-His + 1 M sorbitol + 9 mM 3-AT + 5-FOA and SCD + 5-FOA medium [67]. Desired mutants were those which grew on SCD 5-FOA, but died on SCD-His + 1 M sorbitol + 9 mM 3-AT + 5-FOA. Sixteen candidates were confirmed by serial dilution and α-HA immuno-blot measurement of MAPK pathway reporters. Two mutants that showed strong *SHO1*-dependent mating pathway activation during hyperosmolarity were transformed with the genomic DNA plasmid library (pLEJ009) and plated on SCD-Ura/-Trp medium. For each mutant, ~20,000 transformants were pooled and grown overnight in 20 mL SCD-Ura/-Trp medium, reinoculated in 20 mL fresh SCD-Ura/-Trp medium at A_600_ of 0.1 and grown for 5 h before stimulation with 1 M sorbitol. Cells (~20,000) displaying wild-type eGFP expression levels were collected by FACS (Becton Dickinson Influx^TM^ Cell Sorter, Franklin, NJ, USA) eGFP: 488 nm laser and 530/40 nm bandpass filter; td-Tomato: 561 nm laser and 593/40 nm bandpass filter), and plated on SCD-Ura/-Trp medium. The resulting colonies were pooled and the FACS repeated two additional times. Subsequent colonies were patched onto SCD-Ura/-Trp medium and replica plated onto SCD-Ura/-Trp/-His + 1 M sorbitol + 9 mM 3-AT plates to identify His transformants from which plasmids were isolated and sequenced.

### 2.3. Microscopy of Fluorescent Proteins in Yeast Cells

Mid-exponential phase cells were collected by centrifugation, resuspended in SCD, spread onto a 0.75% agarose pad and imaged under an epifluorescence microscope (Model BH-2, equipped with a 60 × 1.4 numerical aperture [NA] and 100 × 1.3 NA objective lenses; Olympus America, Center Valley, PA, USA). eGFP fluorescence was assessed with a 470 nm (40 nm bandwidth) excitation filter and a 525 nm (50 nm bandwidth) emission filter (Endow GFP 47001; Chroma Technology Corp., Bellow Falls, VT, USA), and td-tomato was imaged using a 560 nm (40 nm bandwidth) excitation filter and a 630 nm (60 nm bandwidth) emission filter (Texas Red 31004, Chroma). The pixel intensities of the HOG (*STL1_prom_*-HA-td-tomato) and mating pathway (*FUS1_prom_*-HA-eGFP) fluorescent reporters were measured in individual cells (*n* = 400–500) and analyzed using CellProfiler™ (Broad Institute, Cambridge, MA, USA) (version 1.0.5122), as described in detail previously [55,68,69].

### 2.4. Cell Extracts and Immunoblotting

Mid-exponential phase cultures (A_600_ = 0.8) were stimulated, as stated, and A_600 nm_ = 1.0 equivalent pelleted by centrifugation. Cell pellets were resuspended in 150 µL ice cold 1.85 M NaOH, 7.5% β-mercaptoethanol, and incubated on ice for 10 min with periodic vortexing. Protein was precipitated by the addition of 150 µL ice cold 50% trichloroacetic acid and incubation on ice for 10 min with periodic vortexing. Precipitated protein was pelleted by centrifugation for 2 min, washed twice with 1 mL cold acetone, dried, and resuspended in 50 µL SDS-PAGE loading buffer [125 mM Tris-Cl (pH 7.5), 5% SDS, 5% glycerol, 0.5% β-mercaptoethanol, 5 µg/mL bromophenol blue]. Samples were boiled for 5 min and then clarified by centrifugation for 5 min at max speed in a microfuge. HA-Rga1 electrophoretic mobility shifts were assessed using 10% acrylamide gels with an acrylamide-to-methylene-*bis*-acrylamide ratio of 75:1. All other protein samples were resolved using standard 10% gels (acrylamide-to-methylene-*bis*-acrylamide ratio of 29:1). Proteins were transferred to nitrocellulose, blocked with Odyssey^TM^ blocking buffer (Licor Biosciences; Lincoln, NE, USA) for 1 h, and probed with the desired antibodies overnight at 4 °C. Immunocomplexes on the blots were visualized by incubation for 2 h with an appropriate infrared dye-coupled secondary antibody diluted 1:10,000 in 1:1 TBS:Odyssey^TM^ block containing 0.1% Tween and 0.02% SDS and, after thorough washing, imaged using an Odyssey^TM^ infrared scanner (Li-cor Biosciences). Antibodies used were: mouse monoclonal α-HA.11 (16B12, Covance; Princeton, NJ, USA) diluted 1:1000 in 1:1 TBS:Odyssey^TM^ block + 0.1% Tween-20; α-Pgk1 [70] diluted 1:10,000 in 1:1 TBS:Odyssey^TM^ block + 0.1% Tween-20; for detection of activated (dually phosphorylated) Hog1, α-phospho-p38 (3D7, Cell signaling Technology; Beverly, MA, USA), and for total Hog1, α-p38 (L53F8, Cell Signaling Technology) diluted 1:500 with 5% BSA in 1X TBS + 0.1% Tween-20; for detection of activated (dually phosphorylated) Fus3 and Kss1, α-phospho-p42/44 (9101, Cell Signaling Technology) diluted 1:250 with 5% BSA in 1X TBS + 0.1% Tween-20; and, α-Fus3 (yC-19, Santa Cruz Biotechnology; Santa Cruz, CA, USA), and for Kss1-(13XMyc)_,_ α-c-Myc mAb 9E10 (Cancer Research Laboratory; UC-Berkeley) diluted 1:1000 and 1:100, respectively, with 5% non-fat milk in 1X TBS + 0.1% Tween-20. Blots probed with the α-phospho-p42/44 antibody were stripped with NewBlot^TM^ Nitro Stripping Buffer (Li-cor Biosciences) for 20 min, washed 3 times for 10 min each time with 1X TBS, and then incubated with the α-Fus3 and α-c-Myc for 1 h at room temp.

### 2.5. Protein Purification and In Vitro Protein Kinase Assays

Hog1-6XHIS, catalytically-inactive Hog1-KD-6XHIS, Hog1-as-6XHIS, and GST-Pbs2-EE proteins were purified and in vitro protein kinase assays with [γ-^32^P]ATP were performed as described previously [55]. GST-3C-Rga1-6XHIS (full-length and shorter fragments) were expressed in *E. coli* strain BL21 (DE3) grown to A_600 nm_ = 0.6 in 1 L LB medium and induced by the addition of 1 mM IPTG for 4 h at 20 °C. Cells were harvested by centrifugation at 7000× *g* for 10 min and washed with 10 mL cold 1X PBS. The resulting pellets were then resuspended in lysis buffer [1X PBS, 1 mM DTT, 0.5% Tween-20, 10% glycerol, 0.2 mg/mL lysozyme] containing EDTA-free Complete Protease Inhibitors (Roche Applied Sciences Inc., Indianapolis, IN, USA), incubated on ice for 30 min and lysed by passage through a French pressure cell at 20,000 PSI twice. Lysates were clarified by centrifugation at 12,000× *g* for 10 min. To the resulting supernatant fraction, 0.5 mL of a 50:50 slurry of glutathione-Sepharose 4B beads (GE Healthcare Inc., Chicago, IL, USA) (prewashed in 5 mL cold 1X PBS) was added and incubated for 1 h at 4 °C. Beads were washed with 3 × 5 mL Protease 3C buffer [50 mM Tris-Hcl (pH 7.0), 150 mM NaCl, 1 mM EDTA, 5 mM β-mercaptoethanol, 0.01% NP-40], followed by the addition of 1 mL Protease 3C buffer and 40 units of PreScission Protease (GE Healthcare Inc.). Proteins were eluted by 3C cleavage at 4 °C for 18 h, and then dialyzed against dialysis buffer [20 mM Tris-Hcl (pH 7.5), 100 mM NaCl, 10% glycerol, 1 mM DTT] overnight at 4 °C.

For kinase assays using the Hog1-as-6XHIS protein and N^6^-furfuryl-ATP-γS [71] (BIOLOG, Breman, Germany), Hog1-as was preactivated by incubation at 30 °C for 30 min with Pbs2-EE and ATP as described previously [55], followed by the addition of 160 µL TBS and 10 µL Ni-NTA beads (Qiagen Inc., Hilden, Germany). After 10 min on ice, the beads were washed 2 × 500 µL with cold TBS. MAPK buffer [50 mM Tris-Cl (pH 7.5), 0.1 mM EGTA, 3 mM MgAc, 1 mM sodium orthovanadate, 1% β-mercaptoethanol] and 1 µg Rga1(339–670) was added along with 1 mM N6-furfuryl-ATP-γS. The 40 µL thiophosphorylation kinase assay proceeded 1 h at 30 °C, and then the protein products were separated by SDS-PAGE and the band corresponding to Rga1 was excised for further analysis.

### 2.6. Immunoprecipitation and Calf Intestinal Phosphatase Treatment of HA-Rga1

Cells pellets (from 40 mL culture) were resuspended in 200 µL cold immunoprecipitation (IP) lysis buffer [40 mM Tris-HCl (pH 7.2), 125 mM potassium acetate, 0.5 mM EDTA, 0.5 mM EGTA, 1 mM DTT, 0.1% Tween 20, 12.5% glycerol, protease inhibitors (Roche Complete EDTA-free protease inhibitor tablets), 1 mM Na_3_VO_4_]. Chilled glass beads were added and the cells were disrupted in a FastPrep 120 (Thermo Fischer Scientific). The lysate was clarified by centrifugation at 16,000× *g* for 5 min at 4 °C. IgG-Sepharose 6 Fast Flow beads (20 µL of 50% slurry) (GE Healthcare Inc.) pre-washed in lysis buffer were added to the lysate and incubated for 1 h at 4 °C and then removed. Fresh pre-washed 50% IgG bead slurry (20 µL) and mouse 4 µg α-HA.11 (16B12) were added. After 1.5 h at 4 °C, beads were washed 2× with phosphatase buffer [100 mM NaCl, 50 mM Tris-HCl (pH 7.9), 10 mM MgCl_2_, 1 mM DTT], suspended in 40 µL phosphatase buffer and split in half. Calf intestinal alkaline phosphatase (CIP; 10 units) (New England Biolabs Inc., Ipswich, MA, USA) was added to one of the aliquots, and both samples incubated at 30 °C for 1 h. After incubation, 4X SDS-PAGE loading buffer was added (final concentration of 1X) and samples boiled 5 min prior to SDS-PAGE and immuno-blotting.

## 3. Results

### 3.1. A Genetic Selection to Recover Mutants that Allow Mating Pathway Activation under Hypertonic Conditions in the Presence of Active Hog1

**Figure 1:**
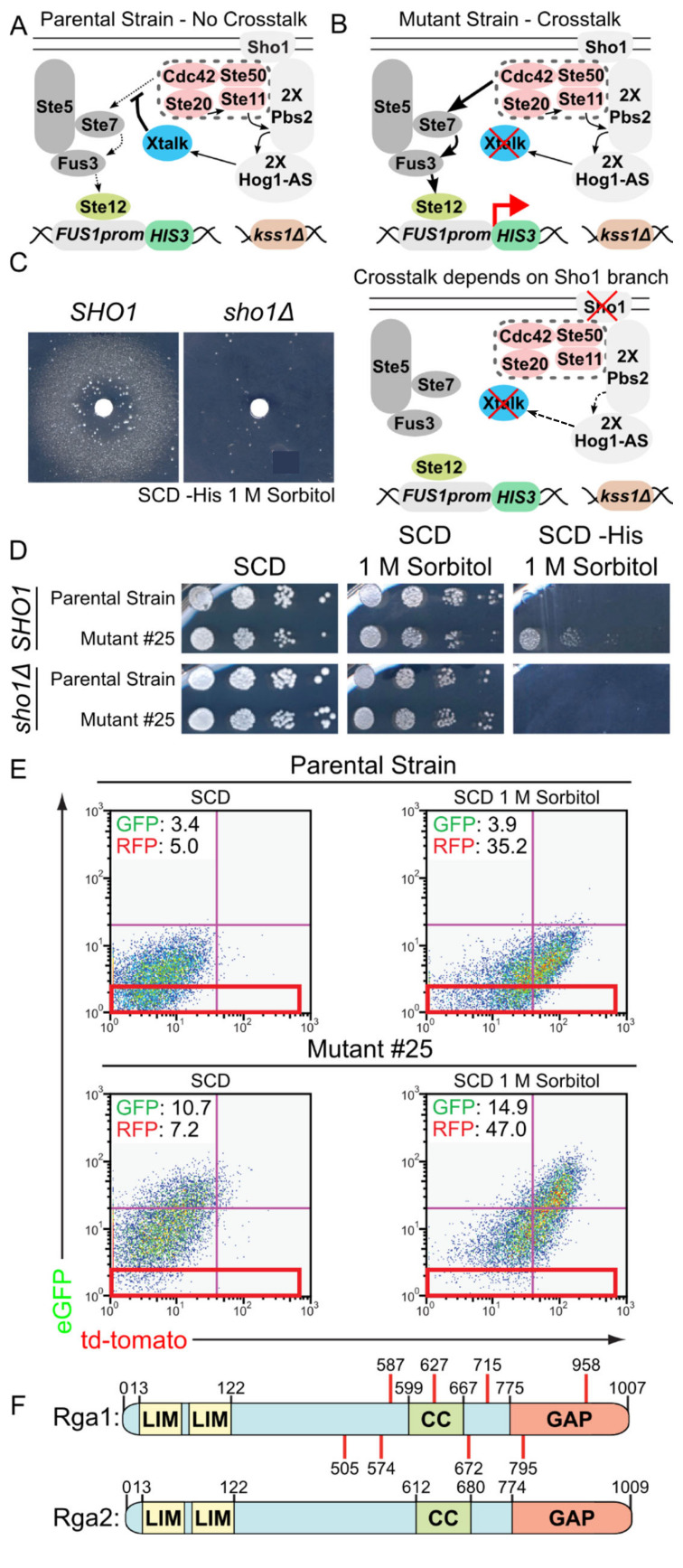
Isolation and identification of a gene required to prevent crosstalk. (**A**) Features of the strain constructed for genetic selection to identify genes required to prevent crosstalk. The Sho1 branch of the HOG pathway shares four signaling components (pink) with the proximal end of the mating pheromone response pathway. Normally, stimulation of the HOG pathway by hyperosmotic shock results only in HOG pathway output (not shown) because activated Hog1 MAPK stimulates, directly or indirectly, the function of some presumed factor(s) (Xtalk; blue) that prevent cross-activation to the mating pathway. (**B**) Expected properties of a mutant deficient in preventing crosstalk. If the Hog1 MAPK is inhibited, stimulation of the HOG pathway cross-activates the mating pathway. Similarly, mutations that impair the function of the presumptive Hog1-regulated factor(s) that blocks inappropriate signaling from the Sho1 branch of the HOG pathway will result in mating pathway output in response to hypertonic stress, as monitored using an appropriate transcriptional reporter (Ste12-responsive *FUS1* promoter-*HIS3* fusion) (top panel). Such mutations should not elicit any cross-activation if Sho1 is absent (bottom panel), because no GTP-bound Cdc42 will be generated via this route. (**C**) Proof of concept. A lawn of the strain used for the genetic selection YJP394 [pRS316-*SHO1*] (*SHO1*), and an otherwise identical strain YJP393 lacking Sho1 [empty pRS316 vector] (*sho1*∆), were plated on solid otherwise complete minimal medium (SCD), but lacking both His and Ura, and containing 1 M sorbitol and 9 mM 3-aminotriazole (3-AT), a competitive inhibitor of His3, thus increasing the threshold of *HIS3* expression required for growth. A sterile filter disk containing 5 µL of a 10 mM stock of the Hog1-as inhibitor 1-NM-PP1 was placed in the center and the plates were photographed after incubation for 4 d at 30 °C. (**D**) Sho1-dependent His^+^ phenotype of a representative mutant. Serial 10-fold dilutions of cultures of YJP394 and Mutant #25, as a representative example, with (top panels) and without (bottom panels) the *SHO1*-containing plasmid, were plated on the indicated media and incubated at 30 °C for 4 d. (**E**) Use of FACS for enrichment of complemented transformants. The parental strain (YJP394) (upper panels) and Mutant #25 (lower panels) transformed with a library of genomic DNA fragments in a *TRP1-* and *KanMX*-marked *CEN* vector were grown to mid-exponential phase in SCD-Ura+G418 and then shifted to fresh medium of the same composition in the absence (left) and presence (right) of 1 M sorbitol. After 2 h, the levels of expression of the fluorescent mating pathway reporter (*FUS1_prom_-eGFP*) and the fluorescent HOG pathway reporter (*STL1_prom_-tdTomato*) were assessed by FACS. Top left corner, median signal intensity for each reporter; red box, region used to define cells expressing eGFP at the basal level (those that were collected). (**F**) Schematic depiction of the primary structure and functional elements in the *S. cerevisiae* Cdc42-GAPs Rga1 and Rga2. LIM, Cys-rich Lin-11, Isl-1 and Mec-3 homology domain; CC, sequence with a continuous 4-3 repeat of hydrophobic residues predicted to have strong propensity to form a coiled-coil; GAP, Cdc42-specific GTPase-activating protein homology domain. Number indicates residue position; red bars, location of the last authentic Rga1 residue in each of the eight unique *rga1* truncations alleles isolated in this study.

To identify proteins that help insulate the HOG pathway from the mating pathway, we constructed a very specific yeast strain (Figure 1A) for isolating novel mutants that fail to prevent crosstalk in a *MAT***a** haploid (Table S1). Because loss of either Hog1 or Pbs2 function allows crosstalk [56], the strain carried two functional copies of *PBS2* and two functional, but analog-sensitive, copies of *HOG1* (*Hog1-as*) [57]. To select for activation of the mating pathway and its MAPK Fus3, these *his3^-^* cells contained a *FUS1_prom_::HIS3* fusion as an integrated transcriptional reporter, in which a Fus3-inducible promoter drives expression of the selectable enzyme (imidazole-glycerol-phosphate dehydratase) marker encoded by the *HIS3* gene (Figure 1B, top) and for which robust *HIS3* expression can be demanded by including in the medium the competitive enzyme inhibitor 3-amino-triazole [72]. The strain also contained an integrated fluorescent reporter for HOG pathway expression (*STL1_prom_::HA-td-Tomato*) and an additional and independent integrated fluorescent reporter for mating pathway expression (*FUS1_prom_::HA-e-GFP*) [55]. To prevent Fus3-dependent cell cycle arrest, and thus allow growth of any His^+^ colonies, the cells carried an allele of Far1 lacking the phosphorylation site (T306A) critical for its function as a CDK1 inhibitor [48,62]. To ensure output dependent on Fus3 alone, the strain also contained a *kss1*∆ deletion. Finally, the strain carried the *SHO1* gene on a *URA3*-marked plasmid (Table S2), so we could readily test in any mutant strain whether mating pathway activation elicited by hyperosmotic stress was generated in a *SHO1*-dependent manner by using 5-fluoro-orotic acid to select for loss of this *URA3*-marked plasmid [67] (Figure 1B, bottom).

As proof of principle, a lawn of this crosstalk detector strain was plated on 1 M sorbitol medium lacking His, and a sterile filter disk containing a compound (1-NM-PP1) that we demonstrated previously to effectively inhibit Hog1-as activity [57] was applied at the center (Figure 1C). As expected, in the zone where the inhibitor concentration was sufficient to reduce (but not totally eliminate) Hog1-as activity, a turbid halo of growth due to crosstalk-induced *FUS1_prom_::HIS3* expression (and sufficient glycerol production to survive on 1 M sorbitol) was observed when *SHO1* was present, but not when it was absent (Figure 1C and Figure S2A).

Having validated the concept, we searched for mutations that are unable to prevent crosstalk by growing multiple independent cultures of the crosstalk detector strain to late exponential phase, plating 10^7^–10^8^ cells of each culture onto separate plates of 1 M sorbitol medium lacking His, and selecting for spontaneously-arising His^+^ colonies. The His^+^ phenotype of each clone was then retested with and without the *SHO1*-containing plasmid. In this way, 16 independent mutants were isolated that grew on 1 M sorbitol medium lacking His in a strictly *SHO1*-dependent manner (Figure 1D shows a representative example) and that clearly elevated the hyperosmotic stress-induced expression of the other independent mating pathway reporter in the same cell, as judged by immunoblotting (Figure S2B shows a representative example). We noted that, generally, the mutants had a somewhat higher basal level of the mating pathway reporters, but expression was always increased upon hyperosmotic challenge. Two mutants (#25 and #31) were chosen for further detailed characterization.

To isolate the wild-type version of the gene presumably mutated in these two candidates, they were transformed with a plasmid-borne yeast genomic DNA library [73], and rare cells in which the crosstalk-induced fluorescent mating pathway reporter expression (*FUS1_prom_::HA-eGFP*) was restored to a normal level, were isolated by multiple rounds of fluorescent-activated cell sorting (FACS) (Figure 1E). Because the mutants have high eGFP expression after hyperosmotic stress, only ~2% of the mutant cells fall within the area of the FACS scatter plot indicated by the red box (Figure 1E, bottom), whereas close to 30% of the parental strain falls within this cut-off (Figure 1E, top). Therefore, a complemented mutant cell should have an expression level that makes it more than 10 times more likely to fall within the red box and should also display a His^-^ phenotype. Thus, performing three rounds of FACS sorting of the transformants, selecting each round for cells falling within the red box, yielded > 1000-fold enrichment for complemented cells, which were also identified by their His^−^ phenotype.

Plasmids were isolated from such complemented His^-^ colonies of both mutants (#25 and #31) and were sequenced. Multiple unique plasmids were recovered from each complemented mutant, based on insert size. Strikingly, however, sequencing showed that the inserts all were derived from chromosome XV and each contained the intact *RGA1* locus, which encodes a Cdc42-specific GAP [74].

Identification of *RGA1* as the complementing gene implied that the two original mutants, and possibly more of the other 14 mutants isolated, should carry mutations at the *RGA1* locus. Consequently, the *RGA1* locus in each mutant was amplified by PCR under high fidelity conditions and sequenced. As anticipated, the two original mutants and the majority of the rest (8 out of 14) contained *rga1* mutations. The 10 *rga1* mutations isolated defined eight unique alleles (Figure 1F, top). Revealingly, each allele truncated Rga1, removing or destroying its C-terminal GAP domain due to the presence of an upstream nonsense codon or a frameshift that led to a nearby nonsense codon (Table S3). *S. cerevisiae* encodes a second, paralogous Cdc42-specific GAP, Rga2 (Figure 1F, bottom) [74]. Rga1 and Rga2 exhibit 31% identity overall, but share significantly greater homology between their N-terminal tandem LIM domains (64% identity) and their C-terminal catalytic (GAP) domains (52% identity) (Figure S3). Aside from Rga1 and Rga2, there are eight other proteins encoded in the *S. cerevisiae* genome with related GAP domains (Bag7, Bem2, Bem3, Lrg1, Rgd1, Rgd2, Rgd3, and Sac7) [18,74,75,76,77]. Of these, only Lrg1 contains N-terminal tandem LIM domains, but all available evidence [76,78,79] demonstrates that it is a GAP for Rho1, not Cdc42. Only Bem3 is thought to be dedicated, like Rga1 and Rga2, solely to Cdc42, but controlling its role in cell morphogenesis (not in signaling per se) [18,74,80]. Moreover, aside from its GAP domain, the large (1128-residue) PX- and PH- domain-containing Bem3 polypeptide is completely divergent in sequence and structure from Rga1 and Rga2. All of the remaining six GAPs appear to be directed against one or more of the five authentic Rho family GTPases (Rho1, Rho2, Rho3, Rho4, and Rho5), not Cdc42 [75,76,77].

### 3.2. A Role for Rga1 in Blocking Crosstalk

**Figure 2:**
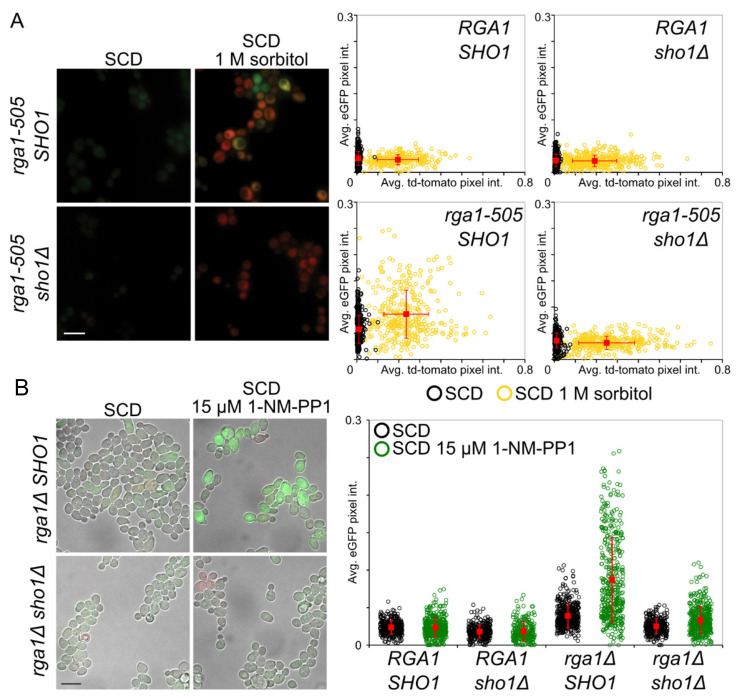
Cdc42-GAP Rga1 is required to squelch crosstalk elicited by hypertonic stress. (**A**) Strains *RGA1 SHO1* (YJP213), *RGA1 sho1*∆ (YJP649), *rga1-505 SHO1* (YJP610), and *rga1-505 sho1*∆ (YJP651) were grown to mid-exponential phase, and stimulated, as indicated, and analyzed by fluorescence microscopy. All four strains contain the HOG-pathway responsive reporter (which induces td-Tomato) and the mating-pathway responsive reporter (which induces eGFP). Quantification of HOG and mating pathway reporter expression (right panels). Each circle in the scatter plot represents the average eGFP (mating pathway reporter) and td-tomato (HOG pathway reporter) pixel intensity for a single cell in either the absence (black dots) or presence (yellow dots) of hyperosmotic stress; whisker plots (red) indicate the population mean and standard deviation. Representative micrographs (left panels) of the *rga1-505* cells, with and without *SHO1*, as indicated, under isotonic conditions (SCD) and after hyperosmotic shock (SCD + 1 M sorbitol). Scale bar (bottom left), 10 µm. (**B**) Cdc42-GAP Rga1 is required to squelch crosstalk elicited by Hog1 inhibition. Strains *RGA1 SHO1* (YJP213), *RGA1 sho1*∆ (YJP649), *rga1*∆ *SHO1* (YJP552), and *rga1*∆ *sho1*∆ (YJP650), all containing *HOG1-AS*, were grown to mid-exponential phase, and then subjected for vehicle only control or 15 µM 1-NM-PP1. Quantification of mating pathway reporter expression only was performed as in (A) (right panels). Representative micrographs of the *rga1*∆ *SHO1* and *rga1*∆ *sho1*∆ cells (left panels. Scale bar (bottom left), 10 µm.

As one means to validate further that Rga1 function contributes to preventing mating pathway activation during hyperosmotic stress, the most severe truncation allele, *rga1-505* (Figure 1F and Table S3), was cloned and integrated as the sole source of Rga1 in otherwise isogenic *SHO1* and *sho1*∆ strains that contained the fluorescent HOG and mating pathway reporters. Unlike the control (*RGA1^+^*) cells, mating pathway reporter expression was markedly elevated in the *rga1-505* mutant cells after hyperosmotic stress, but only in the *SHO1^+^* background, confirming that *rga1-505* was the causative allele and that crosstalk from the HOG pathway was evoking the observed response (Figure 2A). It was noted previously that a *rga1*∆ mutation causes a marked growth debility when combined with either a *hog1*∆ or a *pbs2*∆ mutation, a phenotype that could arise from enhanced crosstalk to the mating pathway and ensuing Fus3-evoked cell cycle arrest, although this possibility was not directly tested [81]. Consistent with that interpretation, others had observed that a *pbs2 rga1* double mutant displayed elevated mating pathway reporter expression under normal growth conditions [82]. As more direct evidence that this behavior is due to elevated crosstalk when Rga1 is absent, we found that inhibition of Hog1-as in *rga1*∆ cells markedly induced mating pathway reporter expression in a *SHO1*-dependent manner even under isosmotic conditions, whereas inhibition of Hog1-as in *RGA1^+^* cells did not (Figure 2B and Figure S4). As measured in the same fashion, inhibition of Hog1-as in *RGA1^+^* cells only results in crosstalk under hyperosmotic conditions [55,57]. Similarly, it was noted previously that *pbs2*∆ *bud14*∆ double mutants display elevated mating pathway reporter expression under normal growth conditions [83]. Bud14 is a regulatory subunit that targets Glc7 (phosphoprotein phosphatase-1/PP1) to the bud cortex [84]. We found that inhibition of Hog1-as in *bud14*∆ cells induced mating pathway reporter expression in a *SHO1*-dependent manner (Supplementary Figure S4), although the level of reporter expression was consistently lower than that in *rga1*∆ cells and there was no measurable further increase upon hyperosmotic shock (Supplementary Figure S5A). Our results demonstrate that the previously reported synthetic genetic interactions between mutations that cripple Hog1 activation and either *rga1* or *bud14* mutations are due to crosstalk to the mating pathway and the ensuing imposition of G1 arrest.

Relative to cells carrying the *rga1-505* allele, a complete null allele (*rga1*∆) exhibited a smaller increase in basal mating pathway reporter expression and no statistically significant increase upon hypertonic shock (Supplementary Figure S5B), suggesting that the inability of *rga1-505* and the other truncation mutations we isolated to prevent crosstalk were not merely due to the loss of its GAP domain function. We suspected that the more severe phenotype of *rga1-505* likely arises because it produces a fragment containing its N-terminal tandem LIM domains, the region of Rga1 that shares the greatest homology with Rga2. Thus, if Rga2 partially compensates when Rga1 is absent, we reasoned that such an N-terminal fragment of Rga1 might act in dominant-negative fashion to interfere with LIM domain-mediated protein–protein interactions necessary for Rga2 localization or function. Consistent with this idea, the characteristics of reporter gene expression of a *rga1*∆ *rga2*∆ double mutant resembled those of *rga1-505* cells, whereas that of an *rga2*∆ single mutant did not (Supplementary Figure S5B). Despite the fact that best estimates indicate that Rga2 (~1900 molecules per cell) is somewhat more abundant than Rga1 (~1300 molecules per cell) [85], our findings indicate that Rga1 is the Cdc42-GAP primarily responsible for preventing crosstalk from the HOG pathway to the mating pathway. When we combined *rga1-505* with a *bud14*∆ mutation, there was a modest additive effect, implying that Rga1 and Bud14 contribute to preventing crosstalk via separate processes. Although multiple mechanisms may be required for complete abrogation of crosstalk [8,9], *rga1-505* clearly had the more pronounced effect in increasing crosstalk to the mating pathway (Supplementary Figure S5A).

**Figure 3:**
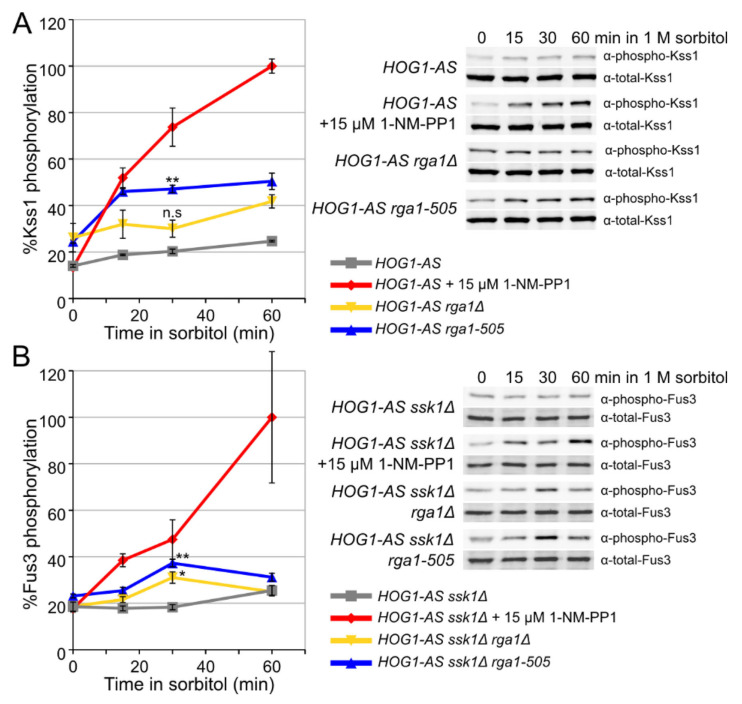
The *rga1-505* mutation permits activation of the mating pathway-associated MAPK Kss1 in response to hyperosmotic stress. (**A**) Cultures of *RGA1^+^* (YJP805), *rga1*∆ (YJP807), and *rga1-505* (YJP813) strains (all carrying the *Hog1-as* allele) were grown, in triplicate, to mid-exponential phase, and then treated with 1 M sorbitol for the indicated amount of time (three *RGA1^+^* cultures were also treated with 1 M sorbitol in the presence of 15 μM 1-NM-PP1, as indicated), and protein extracts were prepared from each time point, resolved by SDS-PAGE, and analyzed by immunoblotting with appropriate antibodies to measure total MAPK and the activated (dually-phosphorylated) species. Representative immunoblots are shown (right panels). The relative band intensities from the triplicate immunoblots of each condition were quantified and plotted (left panel). The highest amount of MAPK activation observed (the ratio of phosphorylated MAPK over total MAPK at any time point) was set at 100%. Statistical significance was assessed using a two-tailed Student’s *t* test, * = *p* < 0.05; ** = *p* < 0.01; n.s., not significant. Error bars represent standard error of the mean. (**B**) The *rga1-505* mutation permits activation of the mating pathway MAPK Fus3 in response to hyperosmotic stress. Protocol and analysis were the same as in (A) for strains *ssk1*∆ *RGA1^+^* (YJP806), *ssk1*∆ *rga1*∆ (YJP808), and *ssk1*∆ *rga1-505* (YJP814) strains (all carrying the *Hog1-as* allele).

As another measure of cross-pathway activation caused by alteration of Rga1 function, we examined the level of activated MAPK using phospho-specific antibodies. As expected, treatment of the cells with 1 M sorbitol alone elicited little or no increase in either activated Kss1 (Figure 3A) or activated Fus3 (Supplementary Figure S6A) due to the lack of crosstalk. As a positive control, the cells, which all express Hog1-as, were treated with 1 M sorbitol in the presence of 1-NM-PP1, which caused robust crosstalk, resulting in readily detectable increases in activated Kss1 (Figure 3A) and activated Fus3 (Supplementary Figure S6A). Compared to the maximal degree of crosstalk achievable by Hog1-as inhibition, the same cells carrying a *rga1*∆ mutation and especially the *rga1-505* mutation also showed a significant increase in activated Kss1 (Figure 3A). Somewhat surprisingly, however, given that our selection was carried out in strains containing Fus3 (but not Kss1), even the *rga1-505* mutation elevated activated Fus3 only rather modestly (Supplementary Figure S6A).

To direct signaling flux solely through the *SHO1* branch, this same analysis was repeated in cells lacking Ssk1, which eliminates the function of the *SLN1* branch of the HOG pathway and should sensitize cells to conditions that promote crosstalk [86]. Indeed, in *ssk1*∆ cells, the effect of the *rga1*∆ and *rga1-505* mutations in elevating the level of activated Fus3 was more pronounced (Figure 3B); however, the duration of Fus3 activation was transient, presumably because, when uninhibited, Hog1 promotes adaptation to elevated osmolarity and, hence, elevated production of GTP-Cdc42 is transitory, and because reversal of Fus3 phosphorylation by MAPK phosphatases also contributes to the observed kinetics [3,87,88]. In any event, these results confirmed that Rga1 makes a significant contribution to preventing crosstalk between the HOG pathway and the MAPKs of the mating and filamentous growth pathways.

### 3.3. Rga1 Negatively Regulates the SHO1 Branch of the HOG Pathway

**Figure 4:**
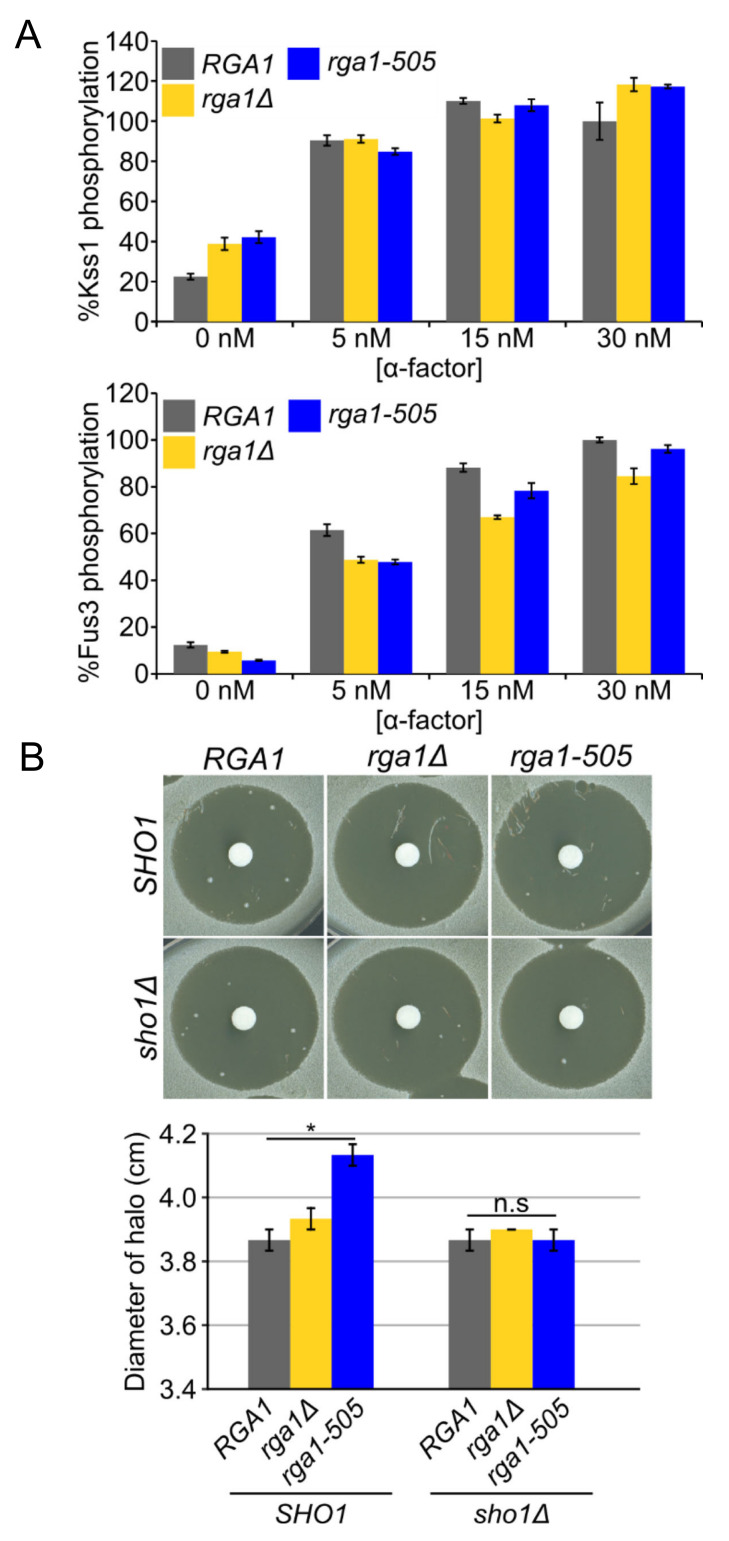
Rga1 does not negatively regulate mating pathway MAPK activation in response to pheromone. (**A**) Strains *RGA1* (YJP805), *rga1*∆ (YJP807), and *rga1-505* (YJP813) were grown to mid-exponential phase, stimulated with the pheromone concentrations indicated for 20 min, and the amount of dually phosphorylated and total MAPK measured by quantitative immunoblotting (for Kss1, see Supplementary Figure S6B; for Fus3, see Supplementary Figure S6C). The amount of activated Kss1 (top panel) and Fus3 (bottom panel) as a percent of the total was calculated for each and then plotted in comparison to the ratio of activated MAPK to total MAPK observed in the control (*RGA1^+^*) strain treated with 30 nM α-factor, which was set as 100%. Experiments were performed in triplicate; error bars, standard error of the mean. (**B**) Apparent sensitization to a pheromone in Rga1-deficient cells arises through basal crosstalk from the HOG pathway. Strains *RGA1* (YJP213), *rga1*∆ (YJP552), and *rga1-505* (YJP610), and their corresponding *sho1*∆ derivatives (YJP637, YJP650, and YJP651 respectively), all of which carry a *bar1*∆ mutation, were grown overnight in YPD. From the overnight cultures, ~2 × 10^7^ cells were mixed in 5 mL of molten top agar (0.75% agarose in YPD, cooled to 45 °C) and immediately plated onto YPD plates. Once solidified, a sterile filter disk was placed on the lawn and 15 µL 1 mg/mL α-factor spotted on the disk. Experiments were performed in triplicate. Representative images (top panel) and plot of mean halo diameter for each strain (bottom panel). Error bars, standard error of the mean. Statistical significance was assessed by a two-tailed Student’s *t* test, * = *p* < 0.05; n.s. not significant.

The fact that loss of Rga1 function was observed to elevate basal mating pathway gene expression [74,82] was construed as evidence for down-regulation of Cdc42 as a negative feedback control within the mating pheromone response pathway itself. However, based on the findings presented in this current study, the observed elevation likely arises via crosstalk from GTP-bound Cdc42 generated in the HOG pathway. To further resolve this issue, we first measured Fus3 and Kss1 activation in *MAT***a** *RGA1*, *rga1*∆, and *rga1-505* strains stimulated with α-factor. Although *rga1*∆ and *rga1-505* mutations increased the basal level of activated Kss1, they did not sensitize either Kss1 or Fus3 activation to lower concentrations of α-factor (Figure 4A and Figure S6B). As judged by a different criterion, the halo bioassay for pheromone-induced growth arrest [89], *rga1*∆ and *rga1-505* did slightly increase the sensitivity of the cells to pheromone action; however, this effect was eliminated in cells lacking Sho1, demonstrating that it depended on crosstalk from the HOG pathway (Figure 4B). Simultaneous stimulation of *rga1-505* cells with both sorbitol and α-factor induced both the HOG and mating pathway reporters with dynamics very similar to those in *RGA1* cells [55], and did not eliminate the modest reduction in mating pathway reporter expression seen during more severe hyperosmotic stress (1 M sorbitol) (Supplementary Figure S7). Most strikingly and revealingly, when over-expressed, *RGA1* reduced mating pathway reporter expression arising via crosstalk (Supplementary Figure S8, 3rd row) much more potently than it reduced mating pathway reporter expression evoked by pheromone (Supplementary Figure S8, 2nd row). Therefore, although Rga1 modestly attenuates signal propagation in the mating pathway, as observed before [82], this effect is due to blocking signal input that arises via crosstalk from the HOG pathway and is not normally an important component of mating pathway down-regulation per se. Therefore, we explored how Rga1 negatively regulates Cdc42 in the HOG pathway.

**Figure 5:**
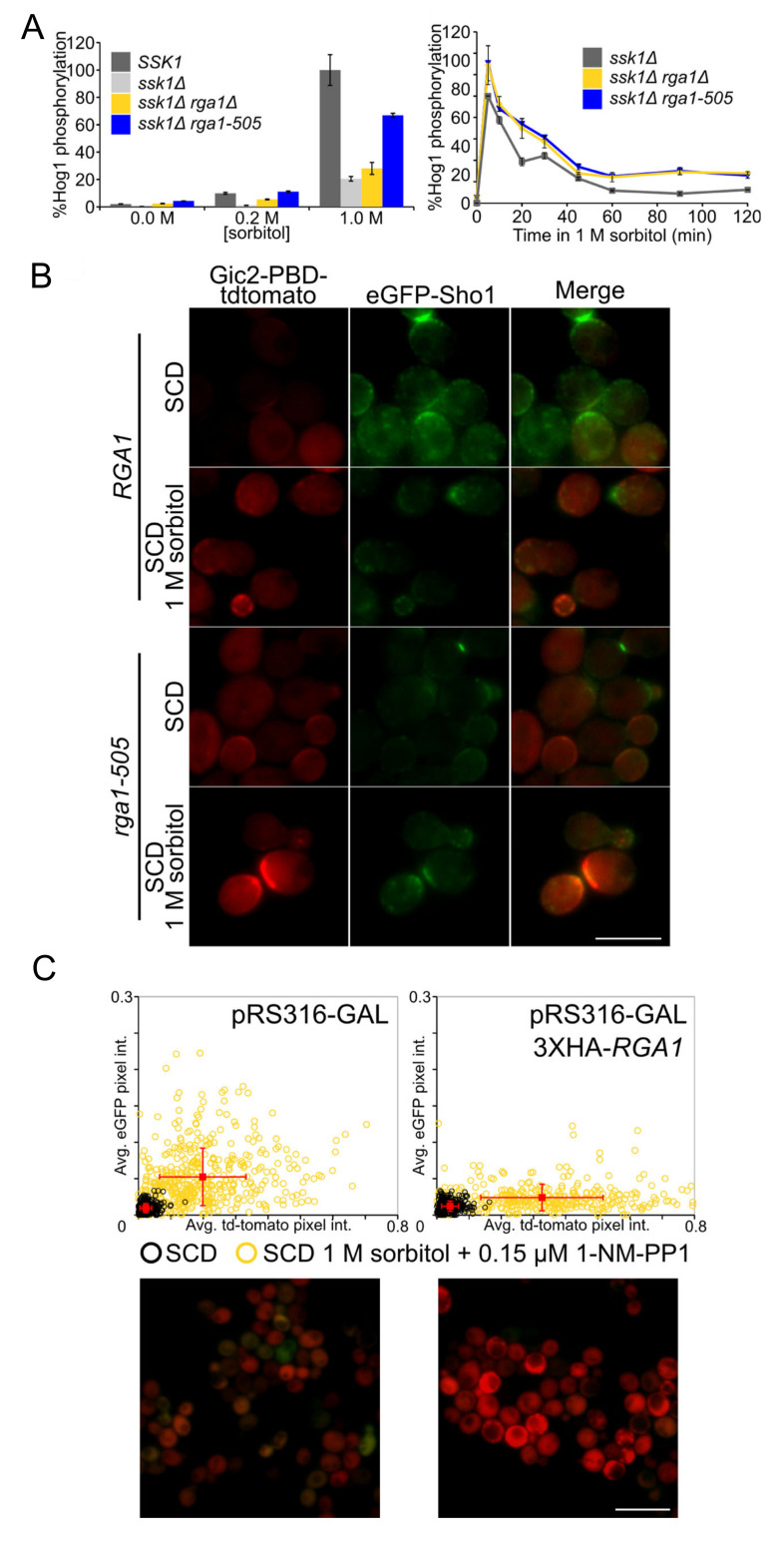
Rga1 negatively regulates the Sho1 branch of the HOG MAPK pathway. (**A**) Strains *RGA1 SSK1* (YJP213), *RGA1 ssk1*∆ (YJP226), *rga1*∆ *ssk1*∆ (YJP591), and *rga1-505 ssk1*∆ (YJP611) were grown to mid-exponential phase, subjected to hypertonic shock for 20 min at the sorbitol concentrations indicated (left panel) and, for the latter three strains, with 1 M sorbitol for the time periods indicated (right panel).The amount of dually phosphorylated and total Hog1 MAPK was measured by quantitative immunoblotting (for sorbitol concentrations, see Supplementary Figure S6D; for time course, see Supplementary Figure S6E). Assays were conducted in triplicate and the amount of activated Hog1 as a percent of the total was calculated for each sample, and then plotted, for the sorbitol dose experiments (left panel), in comparison to the ratio of activated Hog1 to total MAPK observed in the *SSK1^+^ RGA1^+^* strain treated with 1 M sorbitol, which was set as 100%, For the time course (right panels), the amount of activated Hog1 observed at the 5 min timepoint in the *RGA1 ssk1*∆ strain was set as 100%. Error bars, standard error of the mean. (**B**) In Rga1-deficient cells, Cdc42-GTP persists at the plasma membrane. A *rga1*∆ strain carrying a GFP-tagged *SHO1* allele and containing the Cdc42-GTP-binding Gic2-PBD-3xmCherry reporter (YJP654) was transformed with a plasmid (YCplac22) expressing from the *RGA1* promoter either *RGA1* or *rga1-505*. The resulting transformants were grown to mid-exponential phase, collected by centrifugation, and resuspended in 15 µL of SCD medium in the absence and presence of 1 M sorbitol, as indicated. Samples were then spotted onto 0.75% agarose pads, each saturated with the same medium, and viewed under an epifluorescence microscopy within 5–10 min. Representative images are shown. Scale bar (bottom right), 5 µm. (**C**) Elevated expression of Rga1 enhances signaling fidelity. *Hog1-as* (YJP636) cells containing the dual-fluorescent reporters and plasmid pAGL expressing the estradiol-activated GEV (composite Gal4-estrogen receptor-VP16) transcription factor were transformed with either pRS316-*GAL* (empty vector) or pRS316-*GAL-3XHA-RGA1* (pJT4222), as indicated, grown to mid-exponential phase, induced by addition of 20 µM β-estradiol for 2 h, incubated for a further 2 h in the absence or presence of 1 M sorbitol, and then the level of HOG and mating pathway reporter expression were assessed by fluorescence microscopy, quantified in individual cells (top panel), and plotted as in Figure 2A. Representative micrographs of the sorbitol-treated control culture (bottom left) and the sorbitol-treated culture in which *RGA1* was overexpressed (bottom right).

The Sln1 branch of the HOG pathway responds to mild hyperosmotic stress (≤0.2 M sorbitol), whereas activation of the Sho1 branch normally requires more severe hypertonicity (≥0.5 M sorbitol) [90]. If Rga1 action normally reduces flux through the Sho1 branch, then we reasoned that in cells lacking Rga1 their ability to respond to mild hyperosmotic stress should be enhanced. Indeed, in *ssk1*∆ cells in which only the Sho1 branch is functional, an *rga1*∆ mutation and especially the *rga1-505* allele substantially increased the amount of activated Hog1 generated under conditions of both moderate (0.2 M) or severe (1 M) sorbitol treatment (Figure 5A, left, and Supplementary Figure S6D,E). Thus, Rga1 action is a primary cause for the differential sensitivity to mild hypertonic shock of the Sln1 and Sho1 branches. Moreover, Rga1 overexpression effectively squelched HOG pathway reporter gene expression in *ssk1*∆ cells even when subjected to 1 M sorbitol (Supplementary Figure S8, bottom). Kinetic analysis of the hyperosmotic response of *ssk1*∆ cells showed that the level of activated Hog1 achieved in *rga1*∆ cells and especially *rga1-505* cells was higher than *RGA1^+^* cells at every time point (Figure 5A, right). It was also clear that another major influence of the lack of Rga1 was to sustain the level of activated Hog1 over a more protracted period (Figure 5A, right, and Supplementary Figure S6E). Thus, by extension, Rga1 must be important for turning off the Sho1 branch of the HOG pathway during adaptation. In further support of this conclusion, using a fluorescently-tagged derivative of the Cdc42-GTP-binding domain (CRIB) of the cell polarity establishment protein Gic2 as a probe [91,92], we noted that accumulation of GTP-Cdc42 at the PM and colocalizing with Sho1 tagged with GFP was readily observed in *rga1-505* cells, but not in *RGA1^+^* cells (Figure 5B). Moreover, in cells in which crosstalk was elicited by treatment with 1 M sorbitol under conditions where Hog1-as activity was just slightly reduced (only 0.15 μM 1-NM-PP1), overexpression of Rga1 eliminated mating pathway reporter expression (GFP-producing cells were undetectable), and enhanced HOG pathway reporter expression (Figure 5C), reinforcing the conclusion that Rga1 has a role in preventing inappropriate channeling of signaling from the HOG pathway to the mating pathway.

### 3.4. Rga1 Is a Hog1 Substrate In Vitro

Hog1 activity is necessary to prevent crosstalk [55,57]; therefore, Hog1-dependent phosphorylation is required, directly or indirectly, to stimulate processes that block crosstalk. Hog1 phosphorylation of Rga1 itself could provide direct regulation. As for all MAPKs (and CDKs), the preferred consensus site phosphorylated by Hog1 is -SP- or -TP- [93]. Rga1 contains 15 such residues (T149, T278, S291, S331, S445, T470, S519, S521, S529, T532, T541, T571, S679, S749, and T855); strikingly, all of these -SP- and -TP- sites (except T149) were detected as phosphorylated in one or more of the global analyses of the *S. cerevisiae* phosphoproteome [94,95,96,97,98,99,100]. In the presence of purified constitutively-active MAPKK Pbs2 (EE), full-length recombinant Rga1 was phosphorylated in vitro when purified MAPK Hog1, but not a catalytically-inactive Hog1 mutant, was provided (Supplementary Figure S9A). The middle region of Rga1 (residues 340–670) displayed the highest level of Hog1-dependent ^32^P incorporation, presumably because it contains the majority (8 out of 14) of the potential MAPK phospho-acceptor sites detected in vivo (and/or this segment possesses a high-affinity Hog1 docking site). After exhaustive phosphorylation in vitro, the middle region was analyzed by mass spectrometry (MS); the bulk of the incorporation occurred at four TP sites, T470, T532, T541, and T571 (Supplementary Figure S9B), in agreement with the preference of Hog1 for -TP- over -SP- observed using synthetic peptide substrate arrays [93] and in focused surveys of in vivo Hog1 substrates analyzed by mass spectrometry [101,102]. All four sites lie just upstream of a predicted coiled-coil-forming segment in Rga1 (Supplementary Figure S3). In our MS analysis, we also detected phosphorylation, albeit more minor, at quite a number non-consensus sites (Supplementary Figure S9B). The Hog1-as enzyme used for these experiments was purified from yeast to apparent homogeneity, as judged by Coomassie dye staining (Supplementary Figure S6C); reassuringly, multi-dimensional protein identification technology (MUD-PIT) analysis of our purified Hog1-as preparation did not detect the signature peptides for any contaminating protein kinases. We attribute the modification of the minor sites to the conditions we used for exhaustive Hog1-dependent phosphorylation of recombinant Rga1—high enzyme-to-substrate ratio, protracted incubation time, and use of a non-natural phosphoryl donor (N^6^-furfuryl-ATP-γS)].

### 3.5. Hog1 Phosphorylation of Rga1 Is Not Required to Prevent Crosstalk

If the mechanism by which Hog1 action promotes the inhibition of crosstalk is by direct phosphorylation and stimulation of Rga1, then those modifications would presumably be necessary to promote the ability of Rga1 to deactivate GTP-Cdc42. If so, we anticipated that a Rga1 mutant lacking its most prominent Hog1 sites would be unable to prevent crosstalk, like the *rga1-505* allele. For that reason, using site-directed mutagenesis, we constructed a Rga1(T470A T532A T541A T571A) mutant and integrated the DNA sequence encoding it into the genome in place of the normal *RGA1* locus. We found that the resulting strain did not display any elevated crosstalk to the mating pathway (data not shown). We conclude that, although Hog1 is capable of modifying Rga1, direct phosphorylation of Rga1 is not how Hog1 function promotes Rga1-mediated squelching of crosstalk.

### 3.6. Rga1 Undergoes Cell Cycle-Dependent Cdc28/Cdk1-Mediated Phosphorylation

**Figure 6:**
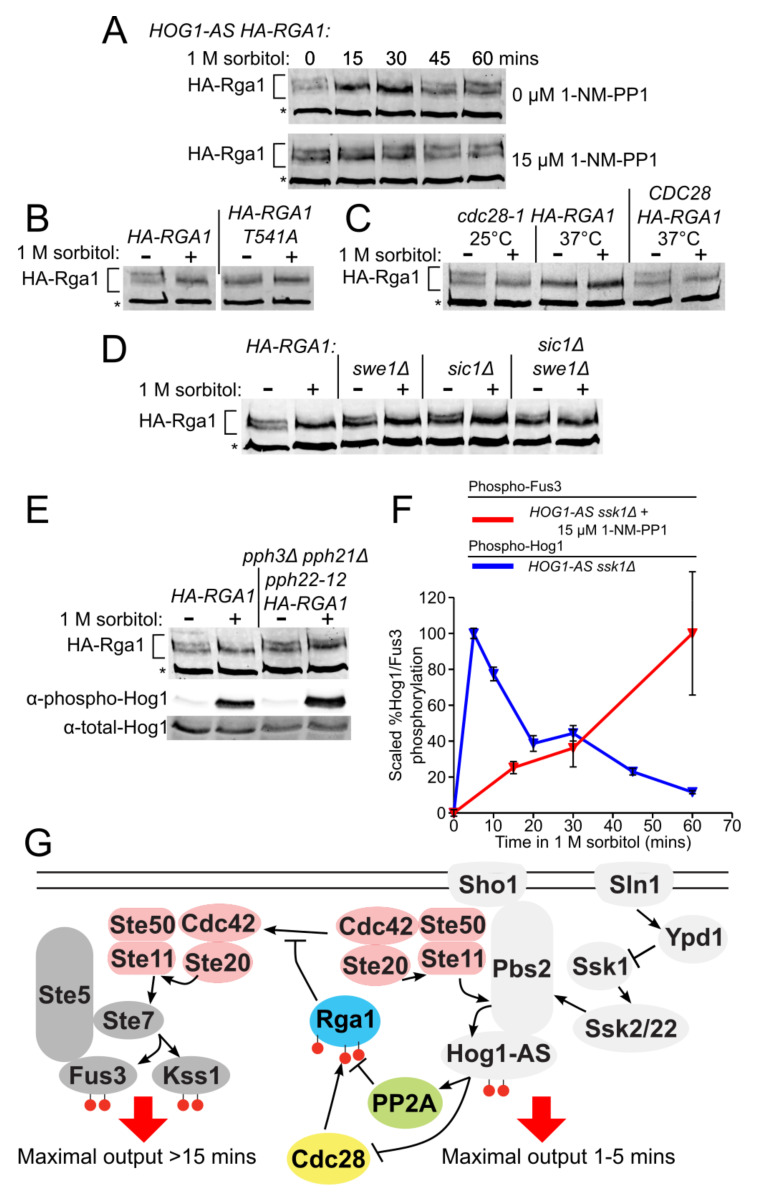
Hog1 action impedes Cdk1-mediated phosphorylation of Rga1 thus promoting its PP2A-dependent dephosphorylation and activation. (**A**) Inhibition of Hog1 maintains Rga1 phosphorylation during hyperosmotic stress. Strain *Hog1-as* (YJP589) containing *HA-RGA1* (pJT3867) was grown to mid-exponential phase and then stimulated with 1 M sorbitol in the presence or absence of 15 µM 1-NM-PP1 for the indicated times, and the electrophoretic mobility of HA-Rga1 was examined by SDS-PAGE and immunoblotting. Asterisk, a non-specific band detected by the α-HA antibody serves as a control for equivalent loading of the lanes. (**B**) Removal of a prominent Cdk1 site mimics the effect of hypertonic shock on Rga1 phosphorylation. Yeast strain *Hog1-as* (YJP589) containing HA-*RGA1* (pJT3867) or *HA-RGA1*(T541A) (pJT3992) were grown to mid-exponential phase, treated with 1 M sorbitol for 15 min, and then examined as in (**A**). (**C**) Rga1 is phosphorylated in a Cdk1 (Cdc28)-dependent manner. Strains *CDC28 HA-RGA1* (YJP679) and *cdc28-1 HA-RGA1* (YJP707) were grown to mid-exponential phase at 25 °C, then either held at that temperature or shifted to 37 °C, as indicated, for 2 h, then treated with 1 M sorbitol for 15 min, and examined as in (**A**). (**D**) Absence of CDK inhibitors Sic1 and Swe1 is not sufficient to restore Cdk1 phosphorylation upon hypertonic shock. Strains WT (YJP694), *swe1*∆ (YJP801), *sic1*∆ (YJP802), and *sic1*∆ *swe1*∆ (YJP803) were grown to mid-exponential phase, then treated with 1 M sorbitol for 15 min and then examined as in (**A**). (**E**) Phosphoprotein phosphatase 2A is required for dephosphorylation of Rga1 after hypertonic shock. Strains *HA-RGA1* (YJP679) and *pph3*∆ *pph21*∆ *pph22-12 HA-RGA1* (YJP755) were grown to mid-exponential phase at 25 °C treated with 1 M sorbitol for 15 min, and then examined as in (**A**). (**F**) Kinetics of Fus3 MAPK activation via crosstalk are significantly slower than Hog1 MAPK activation by hypertonic shock. A *HOG1-as ssk1*∆ strain (JP611) was treated with 1 M sorbitol in the absence of 1-NM-PP1, which elicits Hog1 activation, and in the presence of 1-NM-PP11, which elicits Fus3 activation via crosstalk. The amounts of activated and total Hog1 and Fus3 were determine by SDS-PAGE and immunoblotting, as in Figure 3 and Figure S6. (**G**) A model for Rga1-based crosstalk prevention. Hog1 action prevents crosstalk by stimulating Rga1 function via alleviating its Cdk1-mediated inhibitory phosphorylation.

Because of its large size (113 kDa), it was technically challenging to monitor the phosphorylation status of epitope-tagged Rga1 in vivo by electrophoretic mobility shift; nonetheless, we were able to devise conditions to do so. During exponential growth in normal medium, HA-Rga1 runs primarily as two isoforms of approximately equal intensity (Figure 6A). Within 15 min after exposure to hyperosmotic shock, the slower mobility isoform disappeared with a concomitant increase in the faster mobility isoform, a behavior most consistent with collapse due to dephosphorylation (Figure 6A, top). Consistent with this interpretation, treatment of HA-Rga1 from exponentially-growing cells with calf intestinal phosphatase caused the same collapse (Supplementary Figure S9D). Importantly, when Hog1-as function was blocked with an inhibitor, this apparent dephosphorylation was completely abrogated (Figure 6A, bottom), suggesting that the role of Hog1-dependent phosphorylation is not to modify Rga1 directly, but rather to execute steps that promote Rga1 dephosphorylation. Site-directed mutagenesis indicated that a T541A mutation was sufficient to eliminate the slower mobility species (Figure 6B). The corresponding site (-RT^561^P-) is highly conserved in Rga2 (Supplementary Figure S3) and has been detected as phosphorylated in a global analysis of the *S. cerevisiae* phosphoproteome [100]. Moreover, we noted that the sequence context at this site (-RT^541^P-), i.e., juxtaposed to a basic residue, matches that of three other Cdk1/Cdc28 phosphorylation sites previously mapped in both Rga1 (-KT^278^P-, -KS^291^P- and -HS^331^P-) [95] and in Rga2 (-RT^733^P- and -S^772^PKR-) [95,103]. Indeed, inactivation of Cdc28 by shift of a temperature-sensitive allele (*cdc28-1*) [81] to non-permissive temperature was sufficient to convert HA-Rga1 to the faster mobility species, just like hyperosmotic shock (Figure 6C). Nocodazole synchronization and release experiments indicated that phosphorylation of HA-Rga1 by Cdk1 occurs during G1 (Supplementary Figure S9D).

### 3.7. Phosphorylation of Rga1 by Cdk1 Is Regulated Indirectly by Hog1

Current evidence indicates that the Cdk1-dependent phosphorylation of both Rga1 [104] and Rga2 [105] is inhibitory to their function as Cdc42 GAPs. Collectively, our findings and these prior observations indicate that the primary role of Hog1 action in blocking crosstalk is to counteract the Cdk1-mediated phosphorylation of Rga1. Indeed, it has been reported that Hog1 imposes a transient G1 delay by phosphorylating and stabilizing the CDK inhibitors Sic1 [35] and Cip1 [36], as well as a transient G2 delay by phosphorylating the checkpoint kinase Hsl1, thus preventing its efficient down-regulation of Swe1 [38], a protein kinase that inhibits Cdk1 [106]. This negative regulation of Cdk1 function likely explains why it takes about 45 min after hyperosmotic shock for the mobility of HA-Rga1 to return to its initial Cdk1-dependent pattern (Figure 6A), and is consistent with the most prominent Cdk1-dependent phosphorylation of Rga1 occurring in late G1 (Supplementary Figure S9D).

### 3.8. PP2A Is the Phosphatase that Counteracts the Cdk1-Dependent Phosphorylation of Rga1

We found, however, that hyperosmotic shock still induced the HA-Rga1 mobility shift in *sic1*∆ and *swe1*∆ single mutants and even in a *sic1*∆ *swe1*∆ double mutant (Figure 6D). Hence, Hog1-promoted inhibition of Cdk1 is not the sole mechanism for preventing Cdk1-mediated phosphorylation of Rga1. The most obvious means to achieve the observed hypertonic stress-induced dephosphorylation of HA-Rga1 when Hog1 action has compromised Cdk1 function is the participation of a phosphatase. For this reason, we expressed *HA-RGA1* in all of the mutants in the yeast deletion collection [107] that affect phosphatases and phosphatase regulators. We found that Tpd3, the scaffold (A) subunit of phosphoprotein phosphatase 2A (PP2A), was required for hypertonic stress-induced dephosphorylation of HA-Rga1. Likewise, in a strain lacking two of the PP2A catalytic (C) subunits (*pph21*∆ *pph3*∆) and carrying a hypomorphic allele in a third PP2A catalytic subunit (*pph22-12*) [108], hypertonicity-induced HA-Rga1 dephosphorylation did not occur (Figure 6E, upper panel), even though Hog1 was fully activated (Figure 6E, lower panel). Indeed, even under optimum conditions for inducing Sho1-dependent crosstalk, it requires at least 30 min to see a significant rise in activated Fus3 (Figure 6F); hence, the fact that Rga1 remains dephosphorylated and thus active for at least 45 min when functional Hog1 is present (Figure 6A) provides more than sufficient time for down-regulation of Cdc42-GTP and thus efficient blockade of crosstalk.

In summary, we conclude from our findings that, upon hyperosmotic shock, inhibitory Cdk1-mediated phosphorylation of HA-Rga1 is alleviated and thereby its GAP function activated because activated Hog1 prevents Cdk1-mediated phosphorylation of Rga1, allowing for its efficient PP2A-mediated dephosphorylation. Activated Rga1, in turn, down-regulates the available pool of GTP-Cdc42, preventing crosstalk (Figure 6G).

## 4. Discussion

Here we used a genetic selection to identify Cdc42-GAP Rga1 as a key gene product required to squelch crosstalk between the Hog1 pathway and the Fus3 (and Kss1) MAPK pathways. A chief feature was the ability to screen candidate mutants for the *SHO1* dependence of mating pathway activation by hypertonic stress. Mechanisms for negative feedback within the HOG pathway are invoked to explain how Hog1 action prevents crosstalk, including phosphorylation and down-regulation of Sho1 [56,109] and Ste50 [110,111]. By contrast, Rga1 down-regulates the most upstream component shared by each of these MAPK pathways, consistent with our prior evidence that Hog1 prevents crosstalk by insulating the HOG pathway [55].

By acting on Cdc42-GTP generated locally by the HOG pathway, and because the ability of Cdc42 to diffuse away may be hindered due to association with other proteins in the complex and to its anchoring in the membrane by its geranylgeranylation [112], Rga1 is presumably able to block crosstalk at its source (Figure 6G). In the absence of sufficient GTP-bound Cdc42, Ste20 is inactive and unable to trigger either the mating or filamentous growth MAPK cascades. Ste11, if phosphorylated and activated by Ste20, would require down-regulation by a separate mechanism to suppress crosstalk. Data presented here suggest that the Bud14-bound form of PP1 (Glc7) may fulfill this role.

All things being equal, attenuation of GTP-Cdc42 during hyperosmotic stress would decrease activation of Hog1 and mating pathway MAPKs equivalently. However, even though a reduction in Cdc42-GTP would dampen response through the Sho1 branch of the HOG pathway, the Sln1 branch can maintain Hog1 activation. Moreover, even without a functional Sln1 branch, our results show that the *rga1-505* allele (because it presumably also cripples Rga2 function) increases crosstalk more than it elevates signal flux in the HOG pathway. In *ssk1*∆ cells, 20.3% of the total Hog1 was activated after a 20 min treatment with 1 M sorbitol, whereas 62.6% was activated in *ssk1*∆ *rga1-505* cells under the same condition (Supplementary Figure S6), which is a 3.1-fold increase. By comparison, in *ssk1*∆ cells, the amount of total Fus3 activated after 30 min in 1 M sorbitol did not change appreciably (−0.2% ± 1.1%), whereas 18.9% was activated in *ssk1*∆ *rga1-505* cells (Figure 3B), which is at least a 16.9-fold increase.

In our view, the simplest way to view the function of Rga1 in preventing crosstalk is as a kinetic proof reader [113] (Figure 6G). One classical example of kinetic proof-reading is the 3′-to-5′ exonuclease activity of DNA polymerases, which increases DNA replication fidelity by several orders of magnitude [114]. Discrimination is dictated by the kinetic disparities between the rates of 3′-end strand elongation at a correctly incorporated base versus 3′-end hydrolysis of an inappropriate unpaired base. Likewise, once the HOG pathway is stimulated, maximal activation of Hog1 occurs within 1–2 min, whereas, in the absence of Hog1, Fus3 (and Kss11) activation via crosstalk takes at a minimum at least 10 min [56,57]. Thus, normally, after its activation, Hog1 has ample time to promote the dephosphorylation and activation of Rga1. In turn, activated Rga1 has ample time to promote Cdc42-GTP hydrolysis before this factor can “escape” and encounter Ste20 molecules associated with the signaling machinery, which mediate activation of the Fus3 and Kss1 MAPKs. Although Rga1 negatively regulates a component shared by three MAPK pathways, signal dampening by Rga1 will be greater in those pathways that require more time to become activated after cells are exposed to hyperosmotic conditions. Because GTPases are conserved components of many signaling pathways, it is likely that negative feedback by GAPs will be a mechanism to prevent inappropriate cross-pathway interaction in other situations [115].

One of the most striking observations we made is that even in the absence of any hypertonic shock (i.e., under isosmotic conditions), there was especially potent expression of the mating pathway reporter when the Hog1-as enzyme was inhibited in Rga1-deficient cells. The mating pathway reporter activation that occurred under these conditions was entirely Sho1-dependent. These findings indicate that, normally, even the basal signaling emanating from the Sho1 branch of the HOG pathway is kept in check by the joint actions of Rga1 and Hog1. Thus, when both are removed, crosstalk can occur unimpeded.

Aside from its role in preventing crosstalk, Rga1 seems an important regulator of the Sho1 branch of the HOG pathway, a conclusion also reached by others using a candidate gene approach [116]. As we showed here, in *ssk1*∆ cells carrying a *rga1*∆ and especially a *rga1-505* mutation, response to modest hypertonic shock was almost fully restored, indicating that Rga1 sets the threshold for response by the Sho1 branch. Moreover, as observed by others [95] and confirmed and extended here, Rga1 is clearly a physiologically relevant substrate of Cdk1 (Cdc28); hence, the capacity of the Sho1 branch to respond may be cell cycle-regulated. Consistent with this view, others have attributed ~50% of the variation in HOG pathway reporter expression to cell-to-cell differences, including cell cycle position, rather than to stochastic transcriptional “noise” [117].

In keeping with its multiple roles in vivo, we found phospho-regulation of Rga1 to be complex. Although Hog1 may phosphorylate Rga1 directly, we found that the primary effect of hyperosmotic shock was to cause a Hog1-dependent dephosphorylation of the Cdk1-mediated phosphorylation of Rga1. Moreover, although the transient Hog1-mediated inhibition of Cdk1 described by others may contribute, we found that the resulting slowed rate of Cdk1-mediated phosphorylation simply allowed for more efficient PP2A-mediated dephosphorylation of Rga1. There is no evidence of which we are aware that Hog1 actively stimulates PP2A function; on the other hand, activation of PP2A in mammalian cells by p38, the functional ortholog of Hog1 [118], has been reported [119,120]. Other phosphorylation sites that fit neither the MAPK nor CDK consensus have been identified in Rga1 [121]. How the modification of all these sites affects the activity, stability, localization, and binding partners of Rga1 is a daunting, but ultimately necessary, task to understand its function and dynamics. Nonetheless, we have already demonstrated here that Hog1 activated by hyperosmotic stress impedes crosstalk to the pheromone response pathway via permitting dephosphorylation of inhibitory Cdk1 sites in Rga1.

## 5. Conclusions

How two MAPK pathways that respond to different stimuli, yet share multiple components in common, can generate distinct outputs, has been unclear until now. In this study, we demonstrate that a Cdc42-specific GAP, Rga1, negatively regulates the Sho1 branch of the HOG pathway and also deactivates GTP-bound Cdc42 down to a level insufficient to evoke another Cdc42-dependent MAPK pathway, the mating pheromone response pathway. Thus, this regulatory circuitry blocks inappropriate pathway crosstalk by a kinetic proof-reading mechanism.

## Data Availability

This research did not generate any findings requiring deposition of data in any public repository.

## Supplementary Materials

The following are available online at https://www.mdpi.com/article/10.3390/biom11101530/s1, Figure S1, Schematic depictions of the HOG and pheromone MAPK pathways; Figure S2, Growth and reporter readouts of crosstalk detector strain are Sho1 dependent; Figure S3, Sequence alignment of Rga1 and Rga2; Figure S4, Mating pathway activation during Hog1-as inhibition under isosmotic conditions in *rga1*∆ and *bud14*∆ mutants; Figure S5, Fluorescence microscopy-based analysis of reporter gene expression in *rga1-505* and *bud14*∆ single mutants and in a *rga1-505 bud14*∆ double mutant and in *rga1*∆ and *rga2*∆ single mutants and in a *rga1*∆ *rga2*∆ double mutant; Figure S6, Analysis of the kinetics and dose dependence of Fus3 and Hog1 activation; Figure S7, Analysis of the response of *rga1-505* mutant cells to simultaneous stimulation by increasing concentrations of sorbitol and α-factor; Figure S8, Effect of Rga1 over-expression on reporter gene expression and crosstalk; Figure S9, Analysis of Hog1-mediated Rga1 phosphorylation in vitro and of Rga1 phosphorylation in vivo.
