# Supplementary material for: Cdc42-Specific GTPase-Activating Protein Rga1 Squelches Crosstalk between the High-Osmolarity Glycerol (HOG) and Mating Pheromone Response MAPK Pathways
Source: Biomolecules. 2021 Oct 17;11(10):1530. doi: 10.3390/biom11101530 (PMC8533825; doi:10.3390/biom11101530)
Supplement: Supplementary file 1 [file biomolecules-11-01530-s001.zip › biomolecules-1409432-supplementary.pdf]

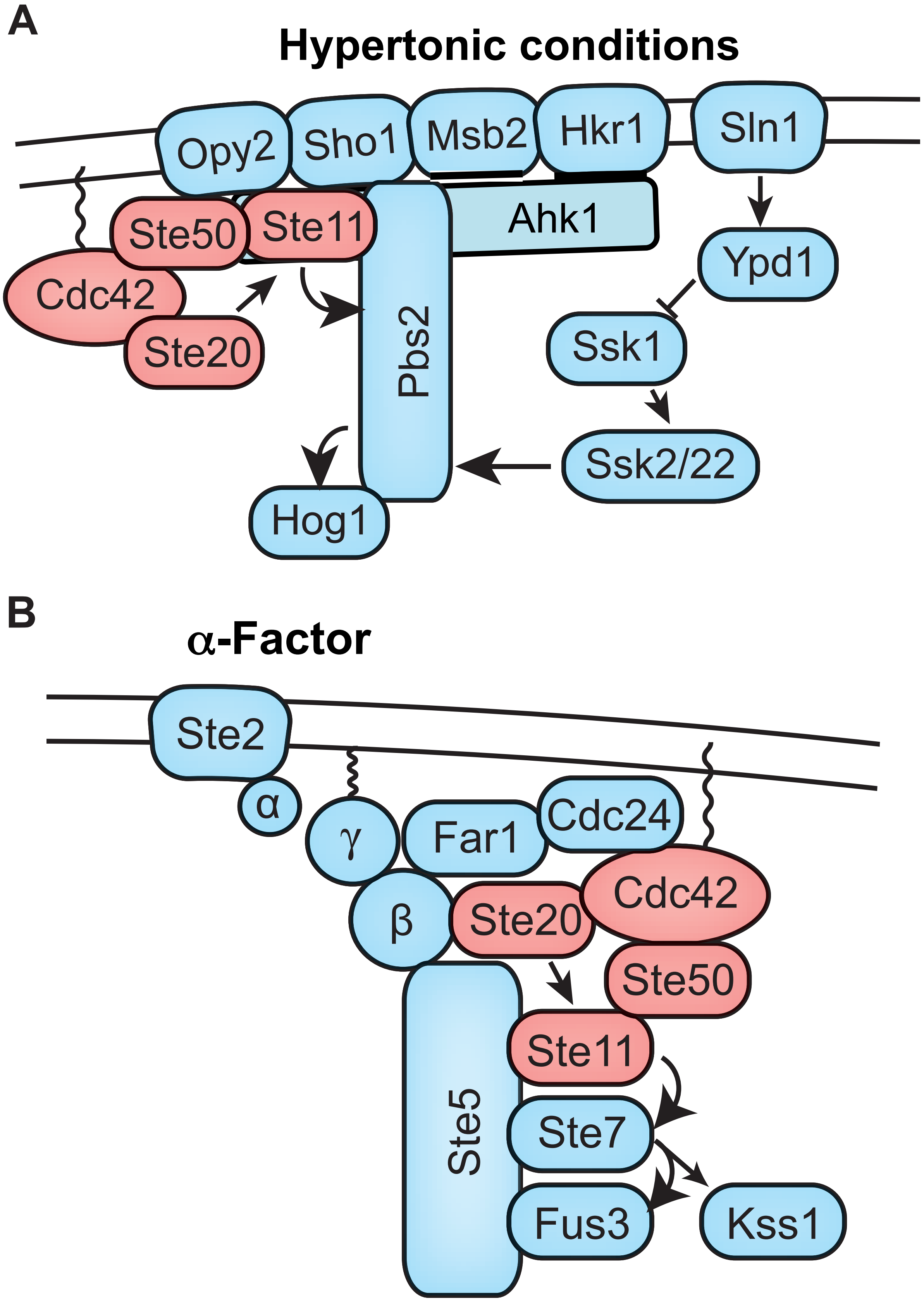


**Supplemental Figure S1**. (**A**) Schematic representation of the *S. cerevisiae* high-osmolarity glycerol (HOG) MAPK pathway. See text of the Introduction for details. (**B**) Schematic representation of the *S. cerevisiae* mating pheromone MAPK pathway. Response of *MAT***a** haploid cells to occupancy of the receptor (Ste2) for α-factor is shown. *MAT*α haploid cells respond to a different pheromone, **a**-factor, through a different receptor (Ste3), but otherwise activate the same downstream pathway. Physical interactions are denoted by overlap or contact of the proteins depicted. Components used by both pathways (*pink*) raise the potential for crosstalk.

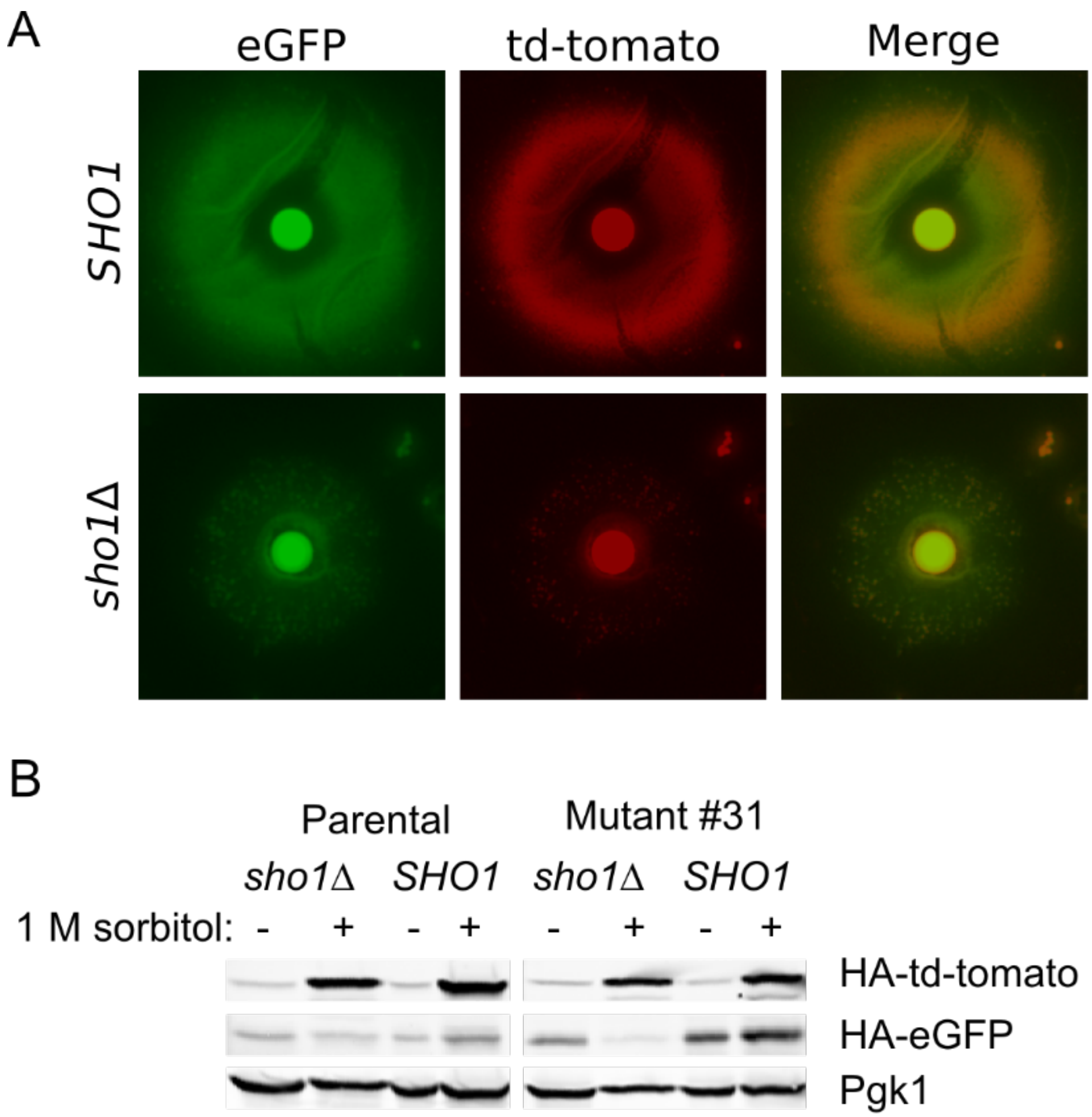


**Supplemental Figure S2**. (**A**) A lawn of the *SHO1*$^+$ strain used for the genetic selection (YJP394) and an otherwise isogenic *sho1*Δ derivative (YJP393) were plated in top agar onto synthetic -Ura/-His medium containing 1 M sorbitol and 9 mM 3-aminotriazole (3-AT), a competitive inhibitor of the His3 enzyme that increases the threshold of *HIS3* expression required for growth. A sterile filter disk was then placed on the lawn and 5 µl of 10 mM 1-NM-PP1 spotted onto the disk to inhibit the Hog1-as present in these cells. After 4 d at 30°C, growth of the cells (assessed by the appearance of turbidity) and expression of the fluorescent HOG pathway transcriptional reporter (*red*) and the fluorescent mating pathway transcriptional reporter (*green*) [assessed by fluorescence imaging of the plates using a Typhoon™ 8600 scanner (Cytiva, Inc., Marlborough, MA)] were examined. (**B**) The parental *SHO1*$^+$ strain ((YJP394) and its *sho1*Δ derivative (YJP393), and a representative mutant (#31) isolated using our genetic selection and its corresponding *sho1*Δ derivative, were grown to mid-exponential phase, collected, resuspended in either fresh medium or the same medium plus 1 M sorbitol. After 2 h, the cells were harvested, lysed and samples of the resulting extracts resolved by standard SDS-PAGE. The level of expression of the HOG (HA-td-Tomato) and mating pathway reporter (HA-eGFP) proteins was assessed by immunoblotting with mouse anti-HA epitope antibodies and infrared dye-labeled secondary anti-mouse antibodies and visualized using a Odyssey™ infrared scanner (Li-Cor, Inc., Lincoln, NE). HA-td-tomato is twice the size of HA-eGFP, permitting their analysis on the same blot. Pgk1 (monitored with rabbit polyclonal anti-Pgk1) served as the loading control.

```
Rga1    MASTAPNEQFPSCVRCKEFITTGHAYELGCDRWHTHCFACYKCEKPLSCESDFLVLGTGA
Rga2    MSADPINDQSSLCVRCNKSIASSQVYELESKKWHDQCFTCYKCDKKLNADSDFLVLDIGT

Rga1    LICFDCSDSCKNCGKKIDDLAIILSSSNEAYCSDCFKCCKCGENIADLRYAKTKRGLFCL
Rga2    LICYDCSDKCTNCGDKIDDTAIILPSSNEAYCSNCFRCCRCSNRIKNLKYAKTKRGLCCM

Rga1    SCHEKLLAKRKYYEEKKRRLKKNLPSLPTPVIDNGHTDEVSASAVLPEKTFSRPASLVNE
Rga2    DCHEKLLRKKQLLLENQTKNSSKEDFPIKLPERSVKRPLSPTRINGKSDVSTNNTAISKN

Rga1    IPSGSEPSKDIETNSSDIVPHFITGYNDSDDNSGSSKFGSNVSIDVIGPEENSTEHVNDD
Rga2    LVSSNEDQQLTPQVLVSQERDESSLNDNNDNDNSKDREETSSHARTVSIDDILNSTLEHD

Rga1    --VKEEAEAPSANMSLNVATDPTLSCKE-PPSHSRN-LLNKTPLRNSSGQYLAKSPSSYR
Rga2    SNSIEEQSLVDNEDYINKMGEDVTYRLLKPQRANRDSIVVKDPRIPNSNSNANRFFSIYD

Rga1    --------------QGIIVND-----------------SLEESDQIDPPNNSSRNASELLTS
Rga2    KEETDKDDTDNKENEIIVNTPRNSTDKITSPLNSPMAVQMNEEVEPPHGLALTLSEATKE

Rga1    VLHSPVSVNMKNPKGSNTDIFNTGEISQMDPSLSRKVLNNIVEETNALQRPVVEVVKEDR
Rga2    NNKSSQGIQTSTSKSMNHVSP-ITRTDTVEMKTSTSSSTLRLSDNGSFSRP---------

Rga1    SVPDLAGVQQEQAEKYSYSNNSGKGRKISRSLSRRSKDLMINLKSRATGKQDSNVKLSPA
Rga2    ------------------------------------QTADNLLPHK----------KVAPSPN

Rga1    SKVTSRRSQDLMRDNDSHTGLDTP-NSNSTS------------------LDILVNNQKSLN
Rga2    KKL-SRSFSLKSKNFVHNLKSKTSEMLDPKHPHHSTSIQESDTHSGWGVSSTHTNIRKSK

Rga1    YKR-FTDNGTLRVTSGK-----------------------------------ETALEEQKNHS
Rga2    AKKNPVSRGQSDSTIYNTLPQHGNFTVPEFNHKKAQSSLGSISKKQNSNDTATNRRINGS

Rga1    FKSPSPIDHLLQSPATPSNVSMYRTPPLDSSLTFDRRNGSSYSNQNYSIPSWQKTPKTQL
Rga2    FTSSSSGHH-----------IAMFRTPPLESGPLFKRPSLSSES--------AHHRSSSLQT

Rga1    ENSDNFEEQKETLYENSESRNDPSLDKEIVTAEHYLKQLKINLKELESQREELMKEITEM
Rga2    SRSTNALLEDDSTKVDATDESATSLEKDFYFTELTLRKLKLDVRELEGTKKKLLQDVENL

Rga1    KSMKEALRRHIESYNSEKNKLYLDSNELSNNPPMINEISLGESPPVKHVATASSVARSSV
Rga2    RLAKERLLNDVDNLTREKDKQSASSRESLEQKENI-ATSITVKSPSSNSDRKGSISNASP

Rga1    KPKFWKFFSSAKP------QTEQSIQGVSTNNTNSIVK---SAPVLLSAPSSGSNSGRLE
Rga2    KPRFWKIFSSAKDHQVGDLESQQRSPNSSGGTTNIAQKEISSPKLIRVHDELPSPGKVP

Rga1    ISPPVLQNPNEFSDVRLVPIENDANMGQSKDGEEYL-DGSNLYGSSLVARCNYENNEIPM
Rga2    LSP----------SPKRL------------------DYTPDGSHLYGSSLQARCAYEKSTVPI

Rga1    ILSVCIDFIESDEENMRSEGIYRKSGSQLVIEEIEKQFSAW-KVQQNTETP--NILTEQD
Rga2    IIRCCIDRIEKDDIGLNMEGLYRKSGSQTLVEEIENEFAQNNSLHSDTISPKLNALLNQD

Rga1    LNVVTGVLKRYLRKLPNPIFTFQIYEPLMRLVKSKKMMENLPFVGGKLSLEAKNSDTYMS
Rga2    IHAVASVLKRYLRKLPDPVLSFSIYDALIDLVRNNQLIERLPLNNDKFLDSPQKVTIYEM

Rga1    SKSALKNILEDLPREHYRVLRVLSEHIEKVTRYSHWNRMTLYNLALVFAPGLIRDFSGEK
Rga2    VLKSLLEIFKILPVEHQEVLKVLAAHIGKVRRCSERNLMNLHNLSLVFAPSLIHDFDGEK

Rga1    DIIDMKERNYIVAFIFGNYKDILT.    1007
Rga2    DIVDMKERNYIVEFILGNYRDIFKQA.  1009
```

**Supplemental Figure S3**. Sequence alignment of *S. cerevisiae* Rga1 and Rga2. Identities, *white letter on a black box*; standard conservative substitutions, *bold letter on a gray box*. Phosphorylated sites shared by Rga1 and Rga2 (*red boxes*) and shown to be phosphorylated (*asterisk*) in global phosphoproteomic analyses cited in the text. Major sites of Hog1-mediated phosphorylation of Rga1 *in vitro* (*blue boxes*) [see also Supplementary Figure S9, Panels A and B). Tandem LIM domains, *underlined in orange*; predicted coiled-coil-forming element, *underlined in black*; catalytic (GAP) domain, *underlined in cyan*. Aligned using NCBI-BLAST at SGD.

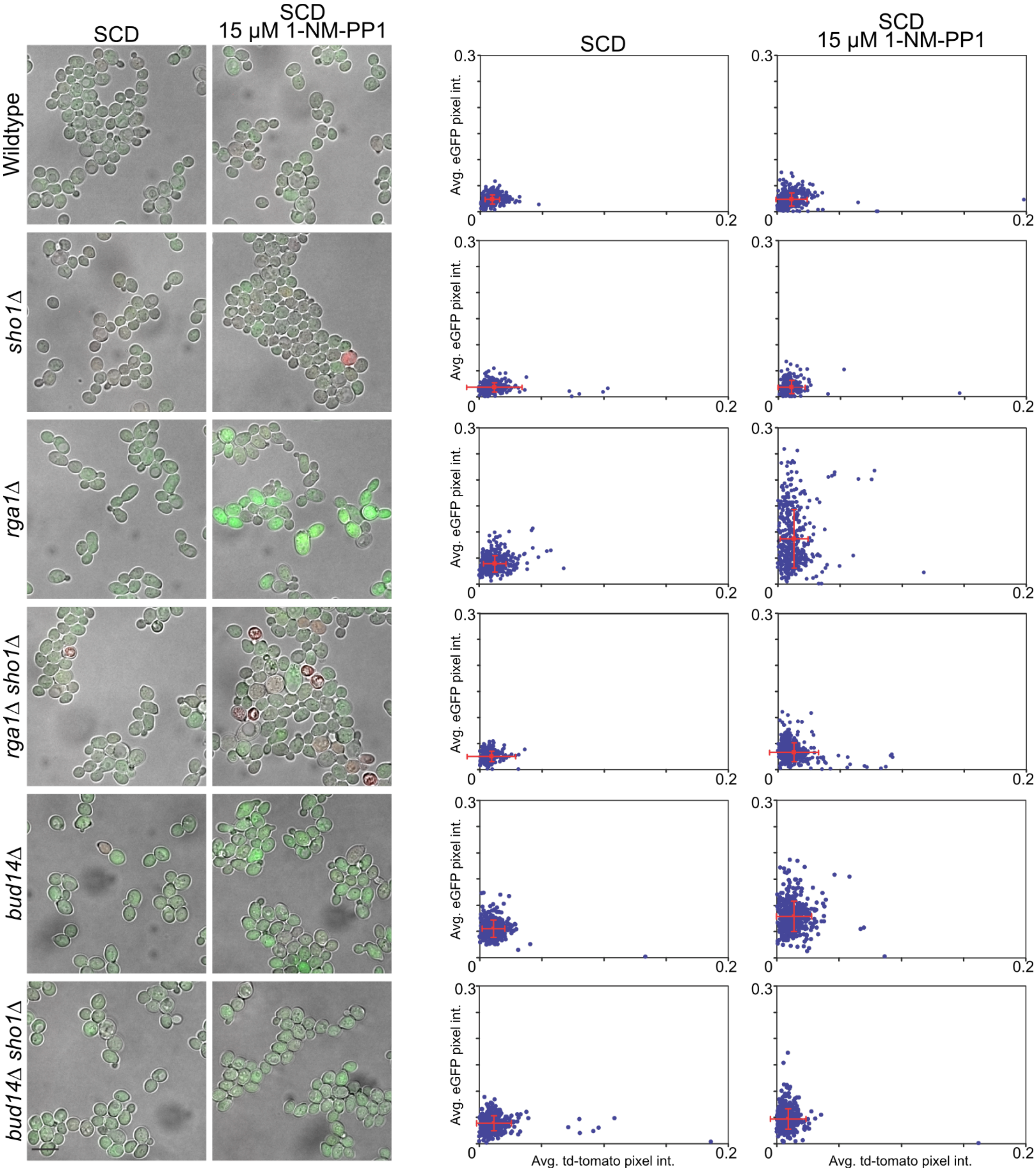


**Supplemental Figure S4**. A *rga1*Δ mutation causes more pronounced crosstalk than a *bud14*Δ mutation upon Hog1 inhibition under isotonic conditions and is strictly dependent on Sho1. Strains YJP213 ("wildtype"), *rga1*Δ (YJP552), and *bud14*Δ (YJP840) and their corresponding *sho1*Δ derivatives (YJP649, YJP650 and YJP841, respectively) were grown to mid-exponential phase and treated in the absence or presence of 15 mM 1-NM-PP1 to inhibit Hog1-as and induce crosstalk. After 2 h, the extent of mating pathway (eGFP) and HOG pathway (td-Tomato) fluorescent reporter expression was assessed by fluorescence microscopy (merged eGFP, td-Tomato and brightfield images; *left panels*) and quantified in individual cells (*right panels*). Scale bar, 10 µm. In the scatter plots, each blue circle indicates the extent of reporter expression in a single cell and the red whisker plot indicates the population mean and standard deviation.

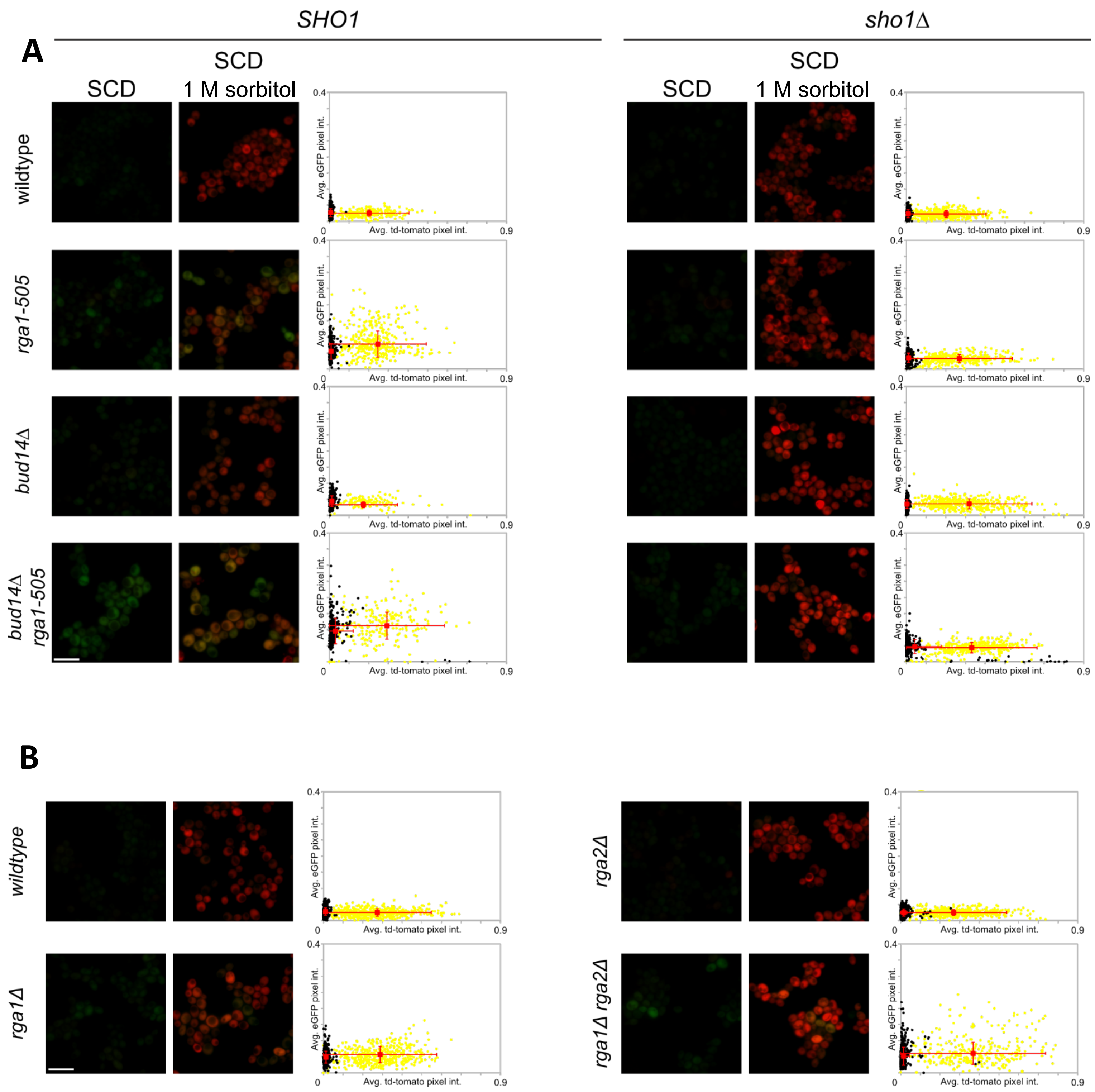


**Supplemental Figure S5**. (**A**) Loss of Rga1 function causes more pronounced crosstalk than loss of Bud14 function in response to hypertonic shock. Strains YJP213 ("wildtype"), *rga1-505* (YJP610), *bud14*Δ (YJP840), and *rga1-505 bud14*Δ (YJP844) and their corresponding *sho1*Δ derivatives (YJP649, YJP651, YJP841, and YJP845, respectively), were grown to mid-exponential phase and a portion of each was left untreated or stimulated with 1 M sorbitol for 2 h. The extent of HOG pathway (td-tomato) and mating pathway (eGFP) fluorescent reporter expression was assessed by fluorescence microscopy (merged eGFP, td-Tomato and brightfield images; *left panels*) and quantified in individual cells (*right panels*), and plotted as in Fig. 2A. Scale bar, 10 µm. (**B**) Effects of loss of Rga1 and Rga2 on crosstalk are additive. Strains YJP213 ("wildtype"), *rga1*Δ (YJP552), *rga2*Δ (YJP585), and *rga1*Δ *rga2*Δ (YJP586) were treated, examined and analyzed, as in (A).

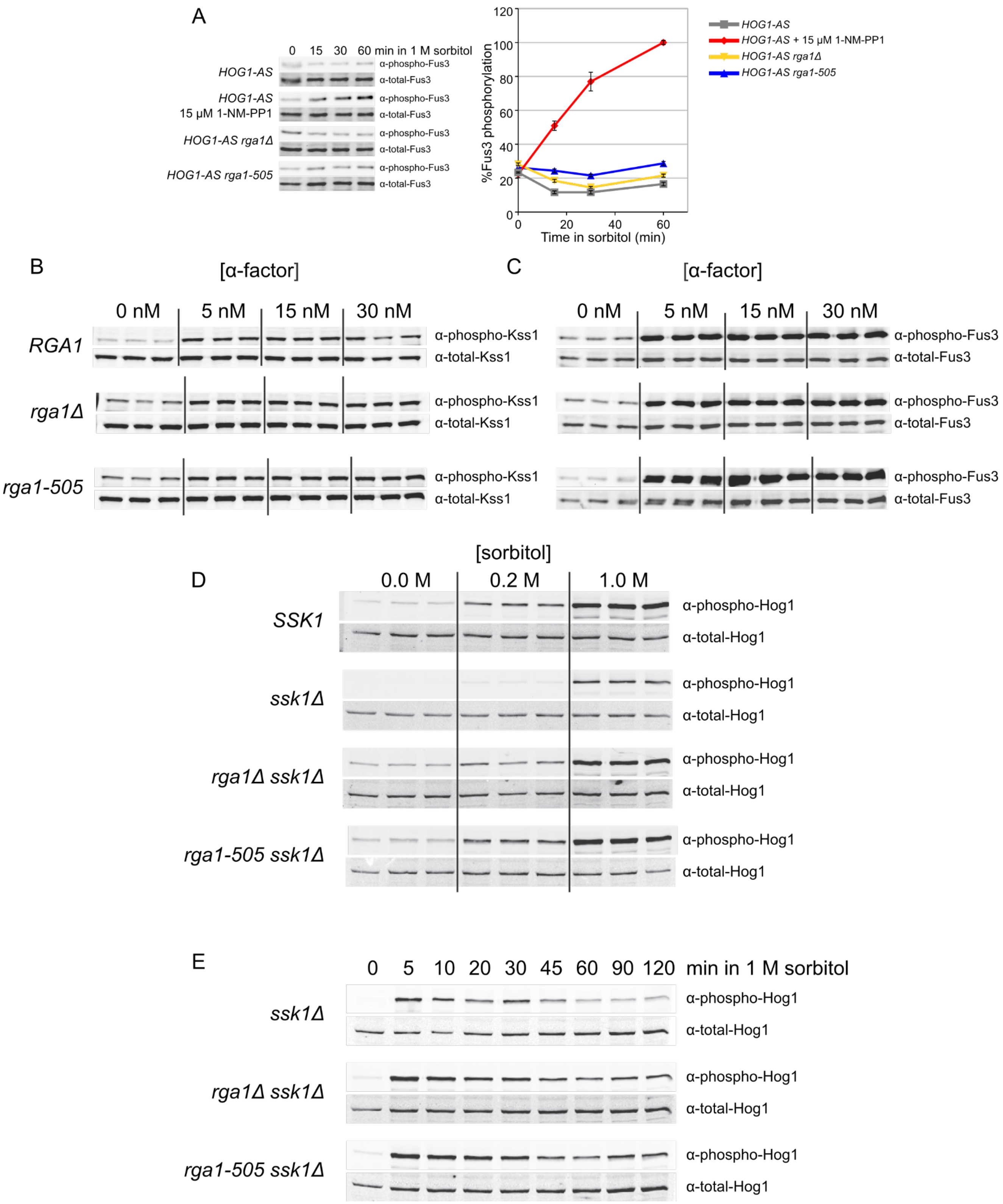


**Supplemental Figure S6**. (**A**) Time course of Fus3 activation via crosstalk in response to hypertonic shock was measured as described for Kss1 in Fig. 3A, using appropriate anti-Fus3 antibodies. (**B**) Assessment of mating pathway MAPK Kss1 activation in response to pheromone. Strains *RGA1* (YJP805), *rga1*Δ (YJP807) and *rga1-505* (YJP813) were grown to mid-exponential phase, stimulated with the dose of pheromone indicated for 20 min, and the amount of dually phosphorylated Kss1 and total Kss1 measured by quantitative immunoblotting. (**C**) Assessment of mating pathway MAPK Fus3 activation in response to pheromone was measured, as in (B), using appropriate anti-Fus3 antibodies. (**D**) Effect of sorbitol concentration on Hog1 activation was assessed as described in Fig. 5A. (**E**) Time course of Hog1 activation was assessed as described in Fig. 5B.

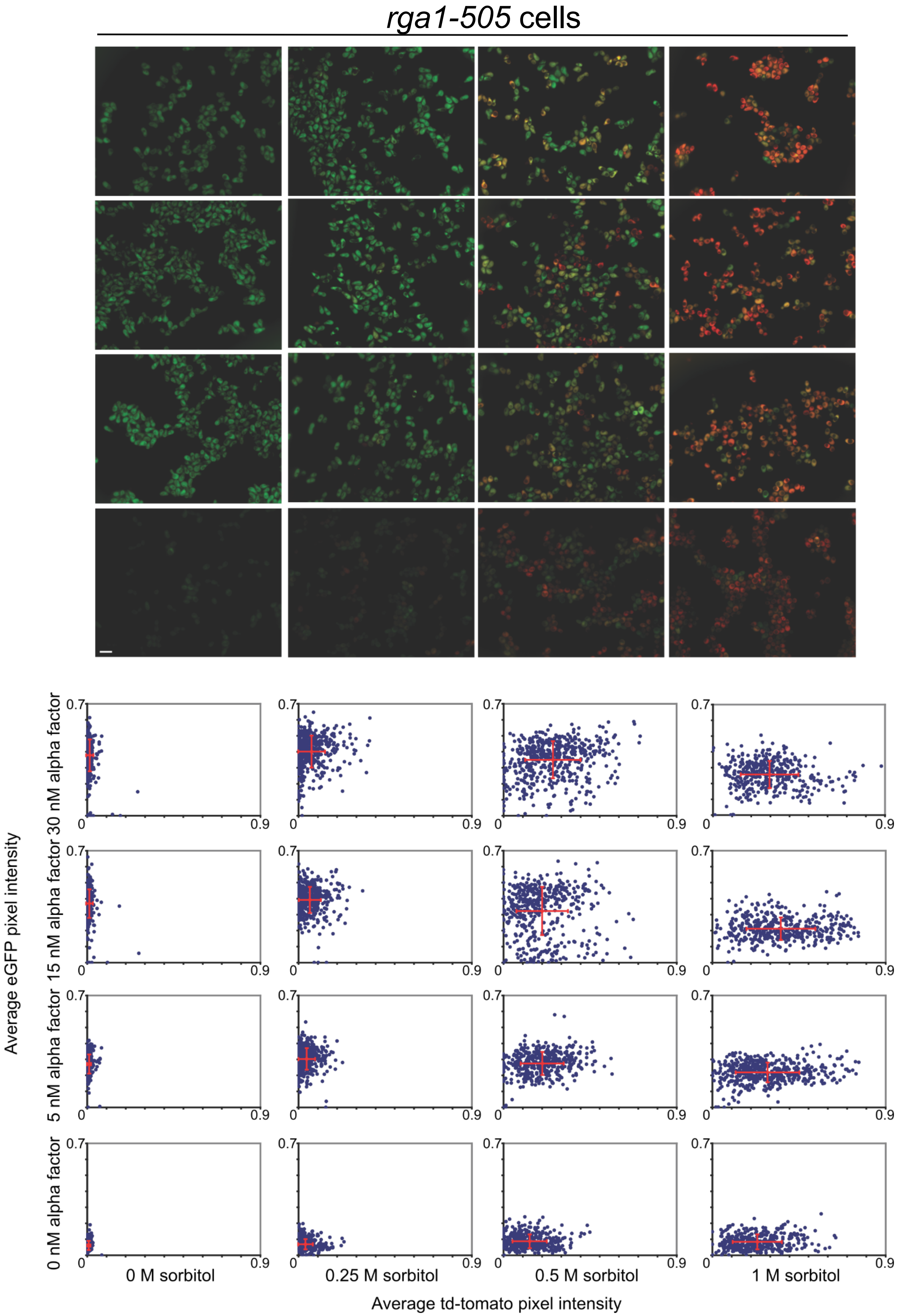


**Supplemental Figure S7**. Response of *rga1-505* mutant cells to simultaneous stimulation with pheromone and sorbitol. Strain (YJP610) was grown to mid-exponential phase, split into equal portions, each of was subjected to concomitant stimulation with the indicated concentrations of $\alpha$-factor and sorbitol. After 2 h, the extent of mating pathway (eGFP) and HOG pathway (td-Tomato) fluorescent reporter expression was assessed by fluorescence microscopy (merged eGFP, td-Tomato and brightfield images; *top panels*) and quantified in individual cells (*bottom panels*), as in Fig. S5.

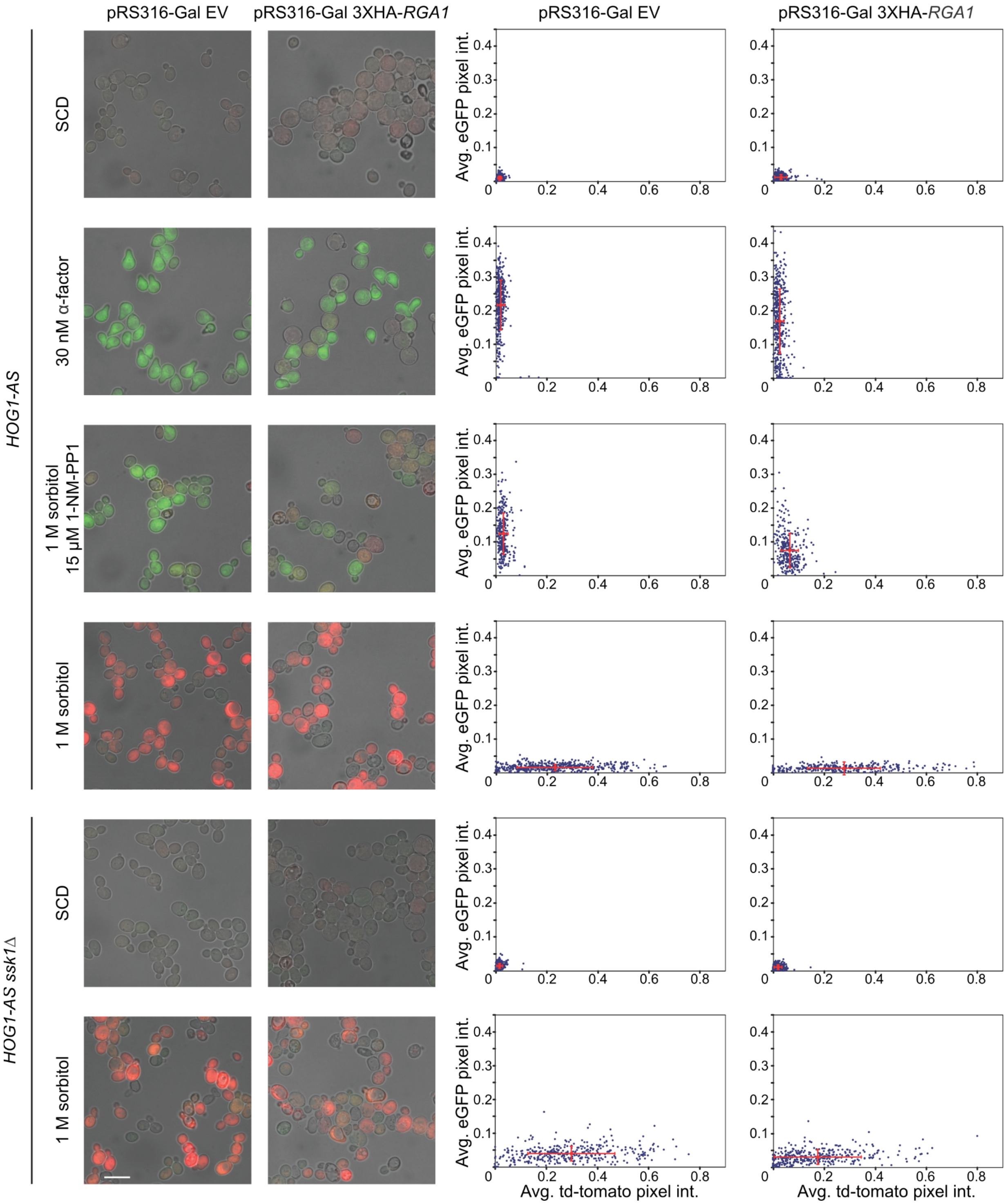


**Supplemental Figure S8**. Overexpression of *RGA1* decreases mating pathway activation evoked via crosstalk more dramatically than mating pathway activation elicited by pheromone. Strains YJP636 (*Hog1-as*) and YJP776 (*Hog1-as ssk1*Δ) containing the dual-fluorescent reporters and an integrated copy of pAGL expressing the estradiol-activated GEV transcriptional activator were transformed with either the empty vector pRS316-*GAL* (EV) or pRS316-*GAL-3XHA-RGA1* (pJT4222). The resulting transformants were grown to mid-exponential phase, treated with 20 μM β-estradiol for 2 h, and then the cells were either not perturbed further or were stimulated with either pheromone (30 μM α-factor), 1 M sorbitol + 15 μM 1-NM-PP1 (to evoke crosstalk), or 1 M sorbitol alone. After 2 h, the extent of mating pathway (eGFP) and HOG pathway (td-Tomato) fluorescent reporter expression was assessed by fluorescence microscopy (merged eGFP, td-Tomato and brightfield images; *left panels*) and quantified in individual cells (*right panels*), as in Fig. S5. The fact that Sho1-dependent HOG pathway output was substantially reduced in the *Hog1-as ssk1*Δ cells served as a positive control to demonstrate that *RGA1* was indeed overproduced under the conditions used.

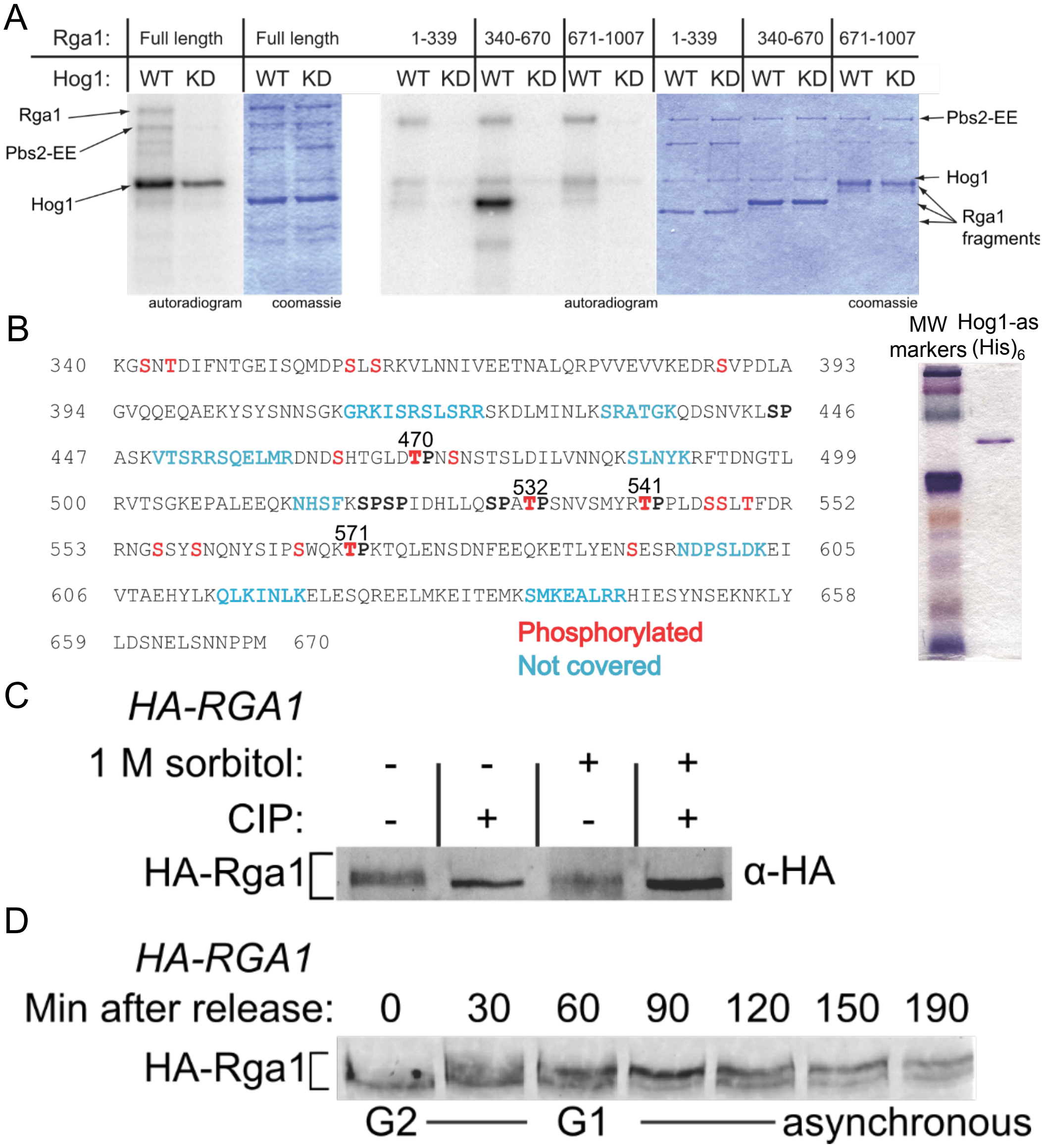


**Supplemental Figure S9**. (**A**) Rga1 is a substrate *in vitro* for purified Hog1 phosphorylated by constitutively-active Pbs2(EE). Wild type (WT) and catalytically-inactive "kinase-dead" (KD) Hog1 purified from yeast (see, Panel B, *right*) were incubated with purified recombinant constitutively-active GST-Pbs2(EE), $[\gamma\text{-}^{32}P]$ATP, and either purified recombinant full-length Rga1 (residues 1-1007) or the indicated Rga1 fragments, as described in Materials and Methods. Reaction products were resolved by SDS-PAGE and radioactivity incorporated into any given species assessed by autoradiography. (**B**) Major sites in Rga1 phosphorylated by purified Hog1. Purified Hog1-as-$(His)_6$ (*right panel*), activated by phosphorylation with Pbs2(EE) and ATP, was incubated with GST-Rga1(340-670) and N6-furfuryl-ATP-γS at 30°C for 1 h. Reaction products were resolved by SDS-PAGE on a 10% gel and the Rga1 band excised, digested with trypsin, and the resulting peptides analyzed for thio-phosphorylated sites by one-dimensional LC MS/MS analysis at the UC Berkeley Vincent J. Coates Proteomics Facility. Primary sites, bold red and numbered (*left panel*). (**C**) Rga1 is phosphoprotein i*n vivo*. Strain (YJP595) expressing HA-Rga1 was grown to mid-exponential phase and equivalent portions of the culture were incubated for 30 min either at a final concentration of 1 M sorbitol (+) or with an equal volume of medium (-). HA-Rga1 was immunoprecipitated from extracts of the resulting cells, incubated in the absence (-) and presence (+) of calf intestinal phosphatase (CIP), as described in Materials and Methods, and then proteins were resolved by SDS-PAGE, and HA-Rga1 detected by immunoblotting. (**D**) Rga1 is phosphorylated in a cell cycle-dependent manner. Strain (YJP679) expressing HA-Rga1 was grown to mid-exponential phase in YPD. To synchronize the cells, DMSO was added to the culture at a final concentration of 1% and, after 10 min of shaking, nocodazole was added to a final concentration of 15 µg/ml. After 90 min, >95% of the culture were large budded cells arrested in the G2 phase of the cell cycle. The cells were released from the cell cycle block by washing them rapidly by two cycles of centrifugation and resuspension in an equal volume of fresh YPD. Immediately thereafter, samples were removed at the indicated times and a portion lysed and processed for immunoblot analysis, as in (C), and a portion was fixed with 3.7% formaldehyde to assess progression though the cell cycle, as determined by changes in morphology monitored by light microscopy.
